# Oligodendrocyte calcium signaling promotes actin-dependent myelin sheath extension

**DOI:** 10.1038/s41467-023-44238-3

**Published:** 2024-01-04

**Authors:** Manasi Iyer, Husniye Kantarci, Madeline H. Cooper, Nicholas Ambiel, Sammy Weiser Novak, Leonardo R. Andrade, Mable Lam, Graham Jones, Alexandra E. Münch, Xinzhu Yu, Baljit S. Khakh, Uri Manor, J. Bradley Zuchero

**Affiliations:** 1 Department of Neurosurgery, Stanford University School of Medicine, Stanford, CA USA; 2 Waitt Advanced Biophotonics Center, Salk Institute for Biological Studies, La Jolla, CA USA; 3 Department of Molecular and Integrative Physiology, Beckman Institute, University of Illinois at Urbana-, Champaign, IL USA; 4 Department of Physiology, David Geffen School of Medicine, University of California, Los Angeles, Los Angeles, CA USA; 5 Department of Cell and Developmental Biology, University of California, San Diego, San Diego, CA USA

**Keywords:** Oligodendrocyte, Actin, Myelin biology and repair

## Abstract

Myelin is essential for rapid nerve signaling and is increasingly found to play important roles in learning and in diverse diseases of the CNS. Morphological parameters of myelin such as sheath length are thought to precisely tune conduction velocity, but the mechanisms controlling sheath morphology are poorly understood. Local calcium signaling has been observed in nascent myelin sheaths and can be modulated by neuronal activity. However, the role of calcium signaling in sheath formation remains incompletely understood. Here, we use genetic tools to attenuate oligodendrocyte calcium signaling during myelination in the developing mouse CNS. Surprisingly, genetic calcium attenuation does not grossly affect the number of myelinated axons or myelin thickness. Instead, calcium attenuation causes myelination defects resulting in shorter, dysmorphic sheaths. Mechanistically, calcium attenuation reduces actin filaments in oligodendrocytes, and an intact actin cytoskeleton is necessary and sufficient to achieve accurate myelin morphology. Together, our work reveals a cellular mechanism required for accurate CNS myelin formation and may provide mechanistic insight into how oligodendrocytes respond to neuronal activity to sculpt and refine myelin sheaths.

## Abstract (web summary)

The cell biological mechanisms that govern myelin sheath extension remain incompletely understood. Here, the authors find that calcium signaling in oligodendrocytes is required for the actin-dependent extension of myelin sheaths.

## Introduction

Myelin, the spirally-wrapped, lipid-rich substance made by oligodendrocytes in the central nervous system (CNS), is critical for the rapid propagation of action potentials by neurons^1^. Myelin loss in diseases like multiple sclerosis or after injury causes severe disability^2^. In addition to its traditional role in increasing conduction velocity, the generation of new myelin and the remodeling of existing myelin sheaths has been increasingly implicated in supporting memory acquisition and learning^3–9^. Normal developmental myelin formation and its remodeling in the context of plasticity are thus essential for a properly functioning nervous system.

To generate myelin, oligodendrocyte precursor cells (OPCs) terminally differentiate and make enormous changes to their cell shape to transform into myelinating oligodendrocytes; these cells must select, ensheath, and then spirally wrap around axons while concurrently longitudinally extending myelin along many different axons. Myelin sheath morphology (e.g. sheath length, myelin thickness) is likely critical for precisely tuning conduction velocity to regulate neuronal network function. During development, sheath length and thickness are dictated, at least in part, by properties of the underlying neurons including axon diameter, neuron type, and neuronal activity (reviewed in^10^). In adults, length and thickness of existing myelin sheaths can be dynamically adjusted by experience or experimentally-induced neuronal activity^3,4,9,11,12^, and these changes are predicted to be sufficient to significantly alter axonal conduction velocity^3,13^. Together, the morphology of myelin sheaths is likely to play a profoundly important role in regulating neural circuit activity. However, how myelin sheath morphology is regulated is still an area of active study.

Myelin is composed mostly of cellular membrane. Because of this, central to myelination is the ability of oligodendrocytes to expand their membranes in precisely the right locations—around and along axons but not out into the neuropil or around non-axonal targets—and to generate sheaths with optimal lengths and thicknesses to support circuit function. To orchestrate the dramatic morphological transformations required for myelin generation, oligodendrocytes employ a myriad of cell biological processes^14^. Membrane addition by exocytosis^15–17^, cytoskeletal dynamics and cell adhesion^18–20^, and external attractive/repulsive cues^21^ all likely collaborate to properly shape myelin sheaths. How are these different cellular processes coordinated to allow myelin sheath morphology to be adjusted to neuronal properties?

Calcium (Ca^2+^) signaling is a likely candidate to regulate oligodendrocyte cell biology during myelin formation and remodeling. In other cell types, calcium signaling plays essential roles in regulating numerous cell biological processes relevant to myelination, including cytoskeletal dynamics, exocytosis, and gene expression^22–26^. Several studies have focused on the roles of calcium signaling in OPCs^27–32^, but fewer have focused on calcium signaling in differentiating oligodendrocytes or during myelination. Oligodendrocytes express numerous calcium-permeable channels and receptors, and calcium transients occur spontaneously and can be induced by a range of neurotransmitters in cultured oligodendrocytes (reviewed in refs. ^32,33^).

Several recent studies have pointed to a potential role of calcium signaling in controlling myelin sheath growth and morphology. In the developing zebrafish, calcium transients occur locally in individual, nascent myelin sheaths, and can be induced by neuronal activity^34,35^. Calcium transients also occur in myelin sheaths in the mouse, both during developmental myelination and during remyelination in adults^36^, although whether these transients are regulated by neuronal activity is still debated^27,36,37^. In zebrafish oligodendrocytes, the pattern of local calcium transients in a nascent sheath predicts whether that sheath will elongate or retract, suggesting that calcium signaling may actively control sheath morphology^34,35^. Consistent with an active role in regulating sheath elongation, whole-cell patch clamping oligodendrocytes with BAPTA to sequester intracellular calcium causes newly-formed sheaths to shorten^35^. However, since BAPTA also directly induces cytoskeletal disassembly in a calcium-independent manner^38^, the causal role of calcium signaling in regulating sheath morphology remains to be determined. Moreover, while the influence of calcium signaling on a diversity of cellular processes is well-studied in other cell types, it remains unknown what cell biological processes calcium may regulate in oligodendrocytes. Since local calcium signaling in myelin sheaths has the potential to bridge neuronal activity to cellular processes capable of remodeling individual sheaths, answering this question may set the stage for future studies to study the mechanism and importance of sheath dynamics during learning.

Here, we used newly-developed genetic tools—the calcium pump “CalEx”^39,40^ and calcium sequestration tool “SpiCee”^41^—to determine the role of oligodendrocyte calcium signaling during myelination of the mouse CNS. Aided by new genetic tools we created to perturb the actin cytoskeleton in specific cell types, we uncover a cellular mechanism used by oligodendrocytes to sculpt myelin sheath morphology: calcium-regulated cytoskeletal assembly in nascent sheaths. This mechanism may explain how myelin sheath geometry is precisely adjusted to neuronal properties during the development and remodeling of neural circuits.

## Results

### CalEx attenuates calcium signaling without affecting oligodendrocyte survival or differentiation

**Fig. 1:**
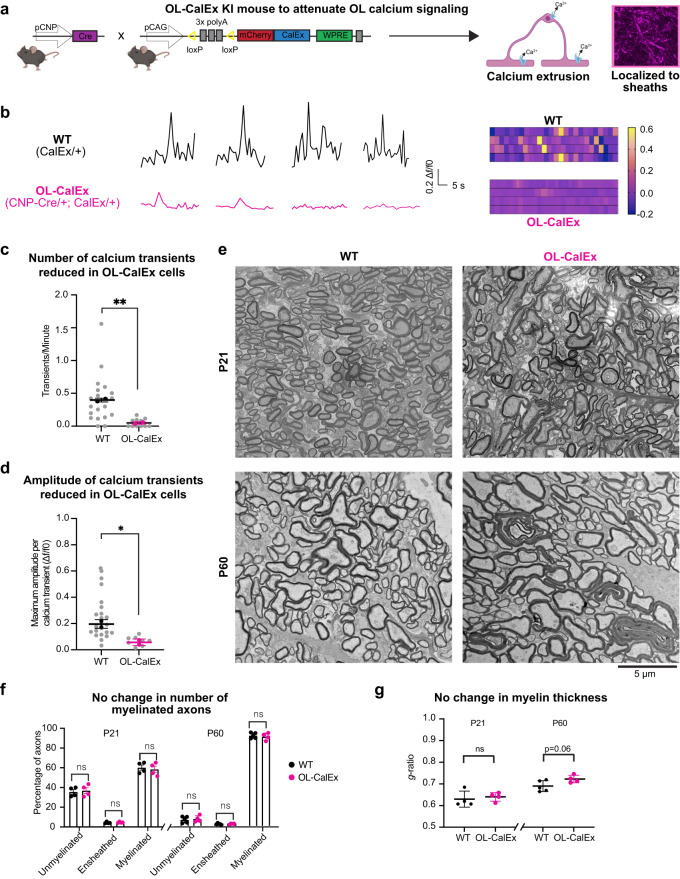
Attenuating oligodendrocyte calcium signaling does not grossly affect oligodendrocyte differentiation or myelination. **a** (left) Genetic strategy for attenuating calcium signaling in oligodendrocytes in vivo. CalEx^flox^ mice (floxed, transcriptional stop cassette in front of human plasma membrane calcium pump, hPMCA w/b) crossed to CNP-Cre. (right) Example of cortical oligodendrocyte expressing mCherry-CalEx. Created with Biorender.com. **b** Example calcium traces and corresponding heatmaps from (top) WT or (bottom) OL-CalEx primary oligodendrocytes. **c** Quantification of number of calcium transients per minute. Mean ± SEM; *N* = 2 biological replicates (preps). Colored bolded dots represent average of individual biological replicates, gray dots represent values from individual cells. Statistical significance determined by unpaired, two-tailed Student’s *t* test; **p = 0.0048. **d** Quantification of calcium transient amplitude in WT and OL-CalEx oligodendrocytes. Average ± SEM; *N* = 2 biological replicates (preps). Colored, bolded dots represent average of individual biological replicates, gray dots represent values from individual cells; *p = 0.0061. **e** (top) Scanning Electron Microscopy of P21 mouse optic nerve cross sections (left: WT, right: OL-CalEx) (bottom) Transmission Electron Microscopy of P60 mouse optic nerve cross sections (left: WT, right: OL-CalEx), Scale bar, 5 μm. **f** Quantification of percentage of unmyelinated, ensheathed, and myelinated axons for P21 and P60 timepoints from electron microscopy in **e**. Average ± SEM, *N* = 4 WT and *N* = 4 OL-CalEx for P21 and *N* = 5 WT and *N* = 4 OL-CalEx for P60. Statistical significance determined by multiple, unpaired, two-tailed Student’s *t* test; n.s. not significant. **g** Quantification of myelin thickness via *g*-ratio for P21 and P60 timepoints from electron microscopy in **e**. Average ± SEM, *N* = 4. Statistical significance determined by unpaired, two-tailed Student’s *t* test; n.s. not significant.

To test the requirement of intracellular calcium signaling in oligodendrocytes during developmental myelination, we used a transgenic mouse model—“CalEx^flox^” (short for Calcium Extrusion)—that allows Cre-dependent expression of a constitutively active plasma membrane calcium pump (hPMCA2w/b) to extrude cytoplasmic calcium in Cre-expressing cells (Fig. 1a)^39,40^. We crossed CalEx^flox/flox^ and Cnp-Cre mice^42^ to attenuate calcium signaling specifically in pre-myelinating cells of the CNS and PNS (hereafter referred to as “OL-CalEx”). OL-CalEx mice and wildtype (WT) littermates were born in normal Mendelian frequencies and survived until adulthood, but OL-CalEx mice were significantly smaller than WT littermates (Supplementary Fig. 1a, b).

We validated CalEx efficacy using calcium imaging of primary oligodendrocytes purified and cultured from OL-CalEx or WT littermates (Fig. 1b–d; Supplementary Fig. 2i). In the absence of neurons, primary oligodendrocytes extend numerous processes that flatten and expand into compacted membrane sheets that preserve key aspects of myelin in vivo, making them a tractable model for studying oligodendrocyte cell biology^20,43^. Primary cultured oligodendrocytes exhibit spontaneous calcium transients locally in their processes^44^, potentially analogous to local calcium transients observed in individual sheaths in vivo^34,35^. Compared to WT oligodendrocytes, CalEx-expressing oligodendrocytes had significantly fewer, lower-amplitude calcium transients (Fig. 1b–d and Supplementary Video 1). CalEx expression did not affect oligodendrocyte survival, membrane expansion, or expression of MBP—a proxy for their differentiation and maturation into mature oligodendrocytes (Supplementary Fig. 2a–f). Thus, CalEx is sufficient to attenuate calcium signaling in cultured oligodendrocytes without grossly perturbing their survival or differentiation.

We next quantified the specificity and penetrance of CalEx expression in oligodendrocytes in vivo in the developing mouse spinal cord during active myelination^15,45^ (Supplementary Fig. 1). As expected, CalEx expression was enriched in the white matter compared to neighboring gray matter (Supplementary Fig. 1c). On a cellular level, CalEx expression in vivo and in culture was apparent in both cell bodies as well as myelin sheaths/membrane sheets (Fig. 1a and Supplementary Fig. 2c–e; see also Fig. 3e below). On average, 93% of CalEx-expressing cells (mCherry + ) were identifiably oligodendrocyte lineage cells (Olig2+; mCherry + ). Of all CalEx-expressing, OL-lineage cells, 75% were mature oligodendrocytes (CC1+; mCherry+), while the remaining 25% mCherry+ cells were premyelinating oligodendrocytes or oligodendrocyte precursor cells (OPCs) (CC1-/Olig2+; mCherry+) (Supplementary Fig. 1e). 85% of all mature oligodendrocytes had detectable CalEx expression (Supplementary Fig. 1f), indicating that CalEx expression is highly penetrant in these mice. Similar to our results showing that CalEx expression does not affect survival or differentiation and maturation in culture (Supplementary Fig. 2a–f), the number of OPCs and differentiated oligodendrocytes was not significantly different between CalEx and WT littermates (Supplementary Fig. 1g–i). Together, these results established CalEx as a precision tool for specifically attenuating oligodendrocyte calcium signaling during myelination.

### Oligodendrocyte calcium signaling regulates myelin membrane morphology during development

What is the consequence of calcium attenuation on myelination? Although calcium transients have been observed in nascent myelin sheaths in vivo^34–36^, it remains unknown whether they have any functional role in myelination. We analyzed myelin ultrastructure in CalEx and WT littermates using electron microscopy on optic nerves harvested during (P21) or at the end of (P60) developmental myelination (Fig. 1e–g). The optic nerve is ideal for quantifying myelination because its small size permits excellent preservation for ultrastructural studies, its axons are aligned, and the time course of myelination has been well-described^20,46–48^. CalEx expression had no effect on the number of myelinated axons at either timepoint (Fig. 1f). Myelin thickness, as quantified by *g*-ratio (the ratio between the inner and outer diameter of the myelin sheath), was also not significantly altered in OL-CalEx mice (Fig. 1g and Supplementary Fig. 3a, b). Additionally, there were no differences in axonal caliber, axonal degeneration, or axonal abnormalities between the two groups at either timepoint (Supplementary Fig. 3c–e).

**Fig. 2:**
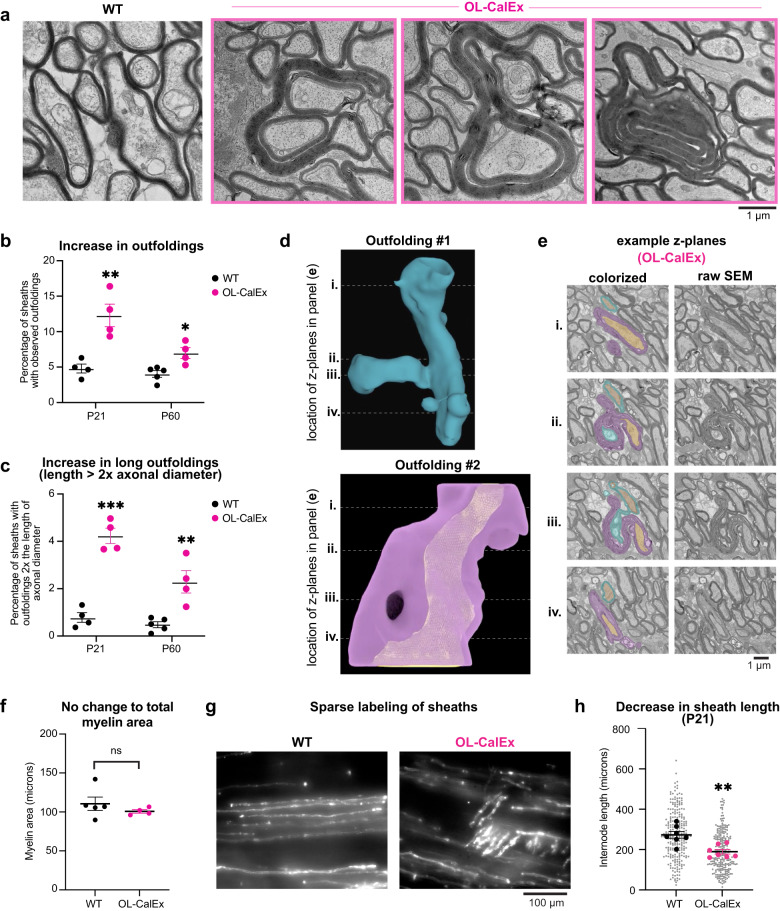
Oligodendrocyte calcium signaling is required for normal sheath length and myelin morphology. **a** Transmission electron microscopy (TEM) of WT (left micrograph) or OL-CalEx (right three micrographs) of P60 mouse optic nerve cross sections, highlighting examples of outfoldings that reach extreme lengths. Scale bar, 1 μm. **b** Quantification of percentages of myelin sheaths with observed outfoldings at P21 and P60 timepoints in **a**. Average ± SEM; P21, *N* = 4 WT and *N* = 4 OL-CalEx; P60, WT *N* = 5, OL-CalEx *N* = 4. Statistical significance determined by unpaired, two-tailed Student’s *t* test; *p = 0.01, **p = 0.0045. **c** Quantification of percentages of myelin sheaths with observed outfoldings greater than 2x the diameter of the axon (which we refer to as long outfoldings) at P21 and P60 timepoints. Average ± SEM; P21, *N* = 4; P60, WT *N* = 5, OL-CalEx *N* = 5. Statistical significance determined by unpaired, two-tailed Student’s *t* test; **p = 0.0044, ***p = 0.0001. Note in **b**, **c** that the actual percentage of sheaths with outfoldings somewhere along their length is much higher than these numbers, which only visualize a small fraction of each sheath (see “Methods” section). **d** 3-dimensional reconstruction of two proximal myelin outfoldings (top, blue; bottom, purple) and their respective axons (orange) from serial sectioning scanning electron microscopy (3DSEM) of P21 OL-CalEx optic nerve. **e** Example (left) colorized and (right) raw scanning EM z planes through 3D reconstructions of both outfoldings. Dotted lines in **d** correspond to four numbered example z-planes through each outfolding. **f** Quantification of total myelin area from P60 TEM in Fig. 1e. Average ± SEM, WT *N* = 5, OL-CalEx *N* = 4; n.s., not significant. **g** Epifluorescence microscopy of P21 WT and OL-CalEx mouse whole-mount spinal cords with AAV- mediated sparse labeling of oligodendrocytes (myelin basic promoter driven EGFP). Scale bar, 100 μm. **h** Quantification of myelin sheath (internode) length from spinal cords in **g**. Average ± SEM, *N* = 7. Statistical significance determined by unpaired, two-tailed Student’s *t* test; **p = 0.0016.

Unexpectedly, OL-CalEx mice had a significant increase in the number of myelin outfoldings—improper outgrowths of myelin away from the axon (Fig. 2a–c). Outfoldings were frequently extremely long and tortuous and extended into the neuropil where they often encircled neighboring myelinated axons or folded back onto themselves (Fig. 2a). Although WT littermates also had outfoldings at a low frequency, the average length of outfoldings was significantly longer in OL-CalEx mice (Supplementary Fig. 3f). We observed outfoldings at both time points (P21 and P60), with a higher frequency at P21 (Fig. 2b, c). Because ultrathin (~60 nm) sections used for EM only sample a small fraction of the entire myelin sheath (mean length approximately 150 μm at P21^49^), our measurements of outfolding frequency per section grossly underestimate the actual frequency of myelin sheaths with outfoldings in OL-CalEx mice^17^ (see below). Neither the presence nor length of outfoldings were dependent on axon caliber (Supplementary Fig. 3g, h), suggesting that outfoldings could arise from any CalEx-expressing myelin sheath.

To further examine the overall frequency and morphology of myelin outfoldings in OL-CalEx mice, we used serial electron microscopy to create 3D reconstructions of individual axon-myelin sheath pairs in optic nerves (Fig. 2d, e and Supplementary Videos 2 and 3). 3D reconstructions revealed that individual myelin sheaths can have multiple outfoldings that originate from their exterior (abaxonal) sides, and outfoldings from neighboring sheaths can closely interact (Supplementary Video 2). Our 3D reconstructions allowed us to approximate the percent of myelin sheaths that have at least one outfolding somewhere along their length (see Methods, Estimation of actual outfolding frequency from electron microscopy images). This analysis suggests that all myelin sheaths in OL-CalEx mice are likely to have at least one outfolding somewhere along their length ( ~ 1.5 outfoldings per sheath on average; see Methods), which is consistent with our 3DSEM reconstructions.

In principle, outfoldings could arise due to either (1) myelin overproduction (hypermyelination) or (2) inaccurate growth of myelin membrane. Myelin outfoldings were not accompanied by overall increase in myelin area (Fig. 2f), suggesting that myelin outfoldings represent inaccurate growth rather than overproduction of myelin. Given the lack of change in myelin area but the increase in outfoldings, we next asked if the longitudinal extension of myelin sheaths along axons was affected in OL-CalEx. We used AAV-mediated sparse labeling of oligodendrocytes in the spinal cord to measure sheath length (internode length). OL-CalEx myelin sheaths were on average ~30% shorter than wildtype (Fig. 2g, h; see also Supplementary Fig. 3i), suggesting that calcium signaling in oligodendrocytes is required for the accurate longitudinal extension of myelin sheaths along axons. Consistent with this hypothesis, we also observed outfoldings in OL-CalEx spinal cords as early as P8 (see also below, Fig. 5e, f), indicating that outfoldings are formed by abnormal growth of myelin during the earliest stages of myelination—not by myelin overproduction over time.

Finally, as an additional test of whether oligodendrocyte-autonomous calcium attenuation caused outfoldings, we employed an orthogonal genetic tool for blocking calcium signaling in cells—the genetic “calcium sponge” SpiCee^41^. SpiCee is a fusion protein containing four calcium-binding sites (two from calmodulin and two from a high-affinity parvalbumin variant) that attenuates cellular calcium signaling by binding and sequestering intracellular calcium^41^, a mechanism distinct from CalEx (Supplementary Fig. 4a). Compared to CalEx, the small size of SpiCee ( ~ 615 bp) allows it to be packaged into adeno-associated virus (AAV) for in vivo delivery. We first validated that SpiCee-mRuby3 attenuated calcium signals in primary cultured oligodendrocytes, compared to control mRuby3 (Supplementary Fig. 4b, c). AAV-mediated expression of SpiCee in newly-formed oligodendrocytes in the developing spinal cord caused a ~3-fold increase in the frequency of myelin outfoldings and a ~ 20-fold increase in long outfoldings (>twice the diameter of the axon) (Supplementary Fig. 4d–g). Combined with OL-CalEx experiments, these results suggested that outfoldings arise due to calcium attenuation specifically within oligodendrocytes.

Together, these data showed that oligodendrocyte calcium signaling is dispensable for myelin initiation but is required instead to accurately sculpt the growth of myelin sheaths to achieve their normal length and morphology.

### Calcium signaling regulates actin filament levels in early-stage oligodendrocytes

How does attenuating calcium signaling in oligodendrocytes cause the myelination defects we observed? A major cellular target of calcium signaling is the actin cytoskeleton^50–54^, and actin dynamics are critical for myelination^19,20^. Intriguingly, oligodendrocyte-specific deletion of proteins that promote actin filament assembly also cause abnormally long outfoldings, including the Arp2/3 complex that directly nucleates actin filaments necessary for early stages of myelination^20^ and N-Wasp^55^, a direct upstream activator of Arp2/3 (see also below, Fig. 6 and ”Discussion” section). Thus, we speculated that outfoldings may arise in CalEx mice due to perturbed actin filament dynamics.

**Fig. 3:**
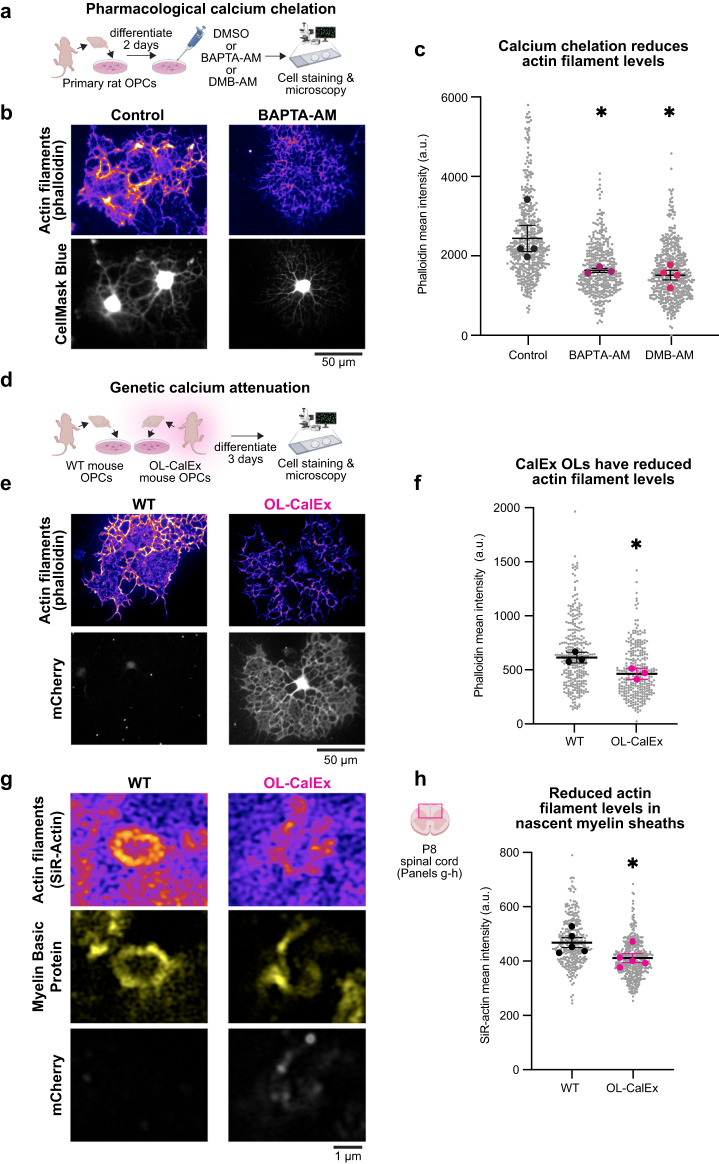
Calcium signaling in early-stage oligodendrocytes regulates actin filament levels. **a** Primary rat oligodendrocyte precursor cells (OPCs) were differentiated for 2 days and then treated overnight with BAPTA-AM, dimethyl-BAPTA-AM (DMB-AM), or DMSO. Cells were then fixed, stained, and imaged. Created with Biorender.com. **b** Epifluorescence representative micrograph of control (left) and BAPTA-AM treated (right) rat oligodendrocytes stained with cell mask blue (white) to label cytoplasmic regions of the cells and phalloidin (purple) to label actin filaments. Scale bar, 50 μm. **c** Quantification of phalloidin mean intensity of rat oligodendrocytes in **c**. Average ± SEM, *N* = 4 biological replicates (preps/bolded dots) p-value was determined by a one-way ANOVA; *p = 0.0188. **d** OPCs were isolated from WT or OL-CalEx mice and differentiated for 3 days before fixation and staining. Created with Biorender.com. **e** Epifluorescence representative micrograph of WT (left) and OL-CalEx (right) mouse oligodendrocytes stained with cell mask blue (white) to label cytoplasmic regions of the cells and phalloidin (purple) to label actin filaments. Scale bar, 50 μm. **f** Quantification of phalloidin mean intensity of mouse oligodendrocytes in **e**. Average ± SEM, *N* = 3 biological replicates (preps/bolded dots). Statistical significance determined by unpaired, two-tailed Student’s *t* test; *p = 0.020. **g** Confocal micrograph of WT (left) and OL-CalEx (right) myelin rings in P8 dorsal spinal cord sections. (bottom row) mCherry staining (middle row) myelin basic protein to label nascent myelin sheaths (top row) SiR-actin staining to label actin filaments. Scale bar, 1 μm. Created with Biorender.com. **h** Quantification of SiR-actin mean intensity in myelin basic protein positive rings. Average ± SEM, *N* = 5. Statistical significance determined by unpaired, two-tailed Student’s *t* test; *p = 0.0499.

To test whether oligodendrocyte calcium signaling regulates actin, we first treated primary rat oligodendrocytes with cell-permeable calcium-chelating drugs (Fig. 3a) and measured their actin filament levels using phalloidin mean intensity in full-cell regions of interest (ROIs)^20^. Actin filament levels were significantly decreased by overnight treatment with either BAPTA-AM (34% reduced) or dimethyl-BAPTA-AM, a BAPTA variant that does not directly disassemble actin^38^ (DMB-AM; 39% reduced; see Methods) (Fig. 3b, c). Next, we purified primary mouse OPCs from OL-CalEx or WT littermates, induced their differentiation into oligodendrocytes, and visualized actin filaments (Fig. 3d). Similar to BAPTA/DMB treatment, actin filament levels were significantly decreased in mCherry-expressing OL-CalEx oligodendrocytes compared WT cells that do not express mCherry (24% reduced) (Fig. 3e, f). Oligodendrocytes in culture and in vivo have a peak of actin filament levels around the time when they are ensheathing axons^20^. Of note, oligodendrocyte actin was only sensitive to calcium attenuation at this exact stage of differentiation (Supplementary Fig. 5g). As with CalEx expression (Supplementary Fig. 2), treatment with BAPTA/DMB had no effect on oligodendrocyte differentiation, cell area, or cell survival for at least five days post-treatment (Supplementary Fig. 5a–f). These results suggested that oligodendrocyte calcium signaling directly regulates actin, rather than indirectly by affecting differentiation.

Does calcium signaling also regulate the oligodendrocyte cytoskeleton in vivo? To look more closely at actin filaments in developing myelin sheaths, we co-stained spinal cord sections for myelin basic protein (MBP) and SiR-actin, a cell-permeable, fluorogenic probe that specifically labels actin filaments^56^ and visualized individual sheaths using Airyscan (super-resolution) microscopy (Fig. 3g). Similar to our culture results, actin filament levels were decreased in (MBP+) nascent myelin sheaths (12% reduced; Fig. 3g, h). This figure may underestimate the actual reduction of actin filaments in nascent sheaths as the resolution limit of Airyscan microscopy (~120 nm laterally) did not permit us to fully exclude SiR-actin signal from axonal actin^57,58^. Together, these data indicate that oligodendrocyte calcium signaling positively regulates actin filament levels in nascent myelin sheaths during development.

### Genetically inducing actin disassembly in oligodendrocytes phenocopies myelin morphology defects observed in OL-CalEx mice

**Fig. 4:**
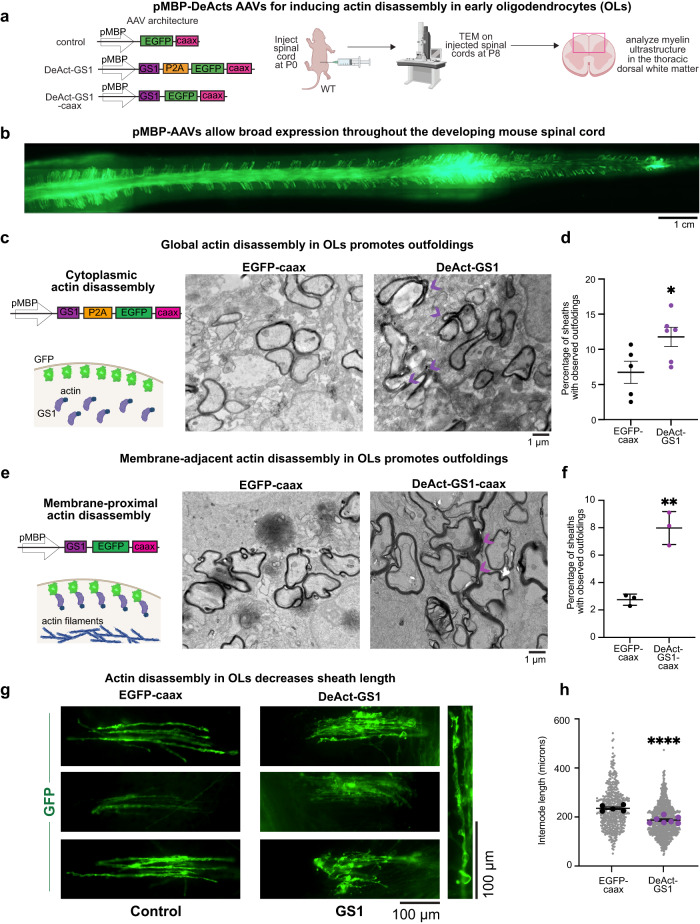
Genetically inducing actin disassembly in oligodendrocytes induces myelin outfoldings and shorter sheaths. **a** (left) Control construct design: myelin basic protein promoter to drive expression in myelinating oligodendrocytes, followed by a farnesylated EGFP for visualization (EGFP-caax). Cytoplasmic DeAct (DeAct-GS1) construct design: myelin basic protein promoter, followed by GS1 segment, followed by P2A self-cleavable peptide, and a farnesylated EGFP for visualization. Membrane-targeted DeAct (DeAct-GS1-caax) construct design: myelin basic protein promoter, followed by a GS1 segment and farnesylated EGFP (one single fusion protein). (right) Virus encoding for DeAct-GS1 or DeAct-GS1-caax or control membrane targeted EGFP were injected into P0 mouse pups. Injected spinal cords were harvested at P8 and processed for transmission electron microscopy. Created with Biorender.com. **b** Whole-mount spinal cord with EGFP expression after injection of AAV-pMBP-EGFP-caax. Shown is 1 representative spinal cord from *N* = 5 injections. Scale bar, 1 cm. **c** Transmission Electron Microscopy of EGFP-caax injected (left) and DeAct-GS1 injected (right) spinal cord sections. Scale bar, 1 μm. Created with Biorender.com. **d** Quantification of percentage of myelin sheaths with observed outfoldings in **c**. Average ± SEM, *N* = 5 EGFP-caax, *N* = 6 DeAct-GS1. Statistical significance determined by unpaired, two-tailed Student’s *t* test; *p = 0.030. **e** Transmission Electron Microscopy of membrane targeted EGFP injected (left) and DeAct-GS1-caax injected (right) spinal cord sections. Scale bar, 1 μm. Created with Biorender.com. **f** Quantification of percentage of myelin sheaths with observed outfoldings in d. Average ± SEM, *N* = 3. Statistical significance determined by unpaired, two-tailed Student’s *t* test; **p = 0.0020. **g** (left) Epifluorescence microscopy of individual oligodendrocytes expressing either EGFP-caax or DeAct-GS1, at P12. (right) Zoom in on individual DeAct-GS1 expressing myelin sheath. Scale bars, 100 μm. **h** Quantification of myelin sheath (internode) length from P12 spinal cords in **g**. Average ± SEM, *N* = 5 EGFP-caax, *N* = 7 DeAct-GS1. Statistical significance determined by unpaired, two-tailed Student’s *t* test; ****p = 0.0001.

Our results thus far predicted that myelin morphology defects may arise in OL-CalEx mice as a result of perturbed actin assembly in oligodendrocytes at the start of myelination. To test this idea, we used DeActs, genetically encoded tools we developed to selectively induce actin disassembly in specific cell types in vivo^59^. We designed an oligodendrocyte-specific DeAct adeno-associated virus (AAV) construct using the myelin basic protein promoter (pMBP)^60^ to restrict DeAct expression to newly formed and mature oligodendrocytes^15^. To label DeAct-expressing myelin sheaths, this construct also expresses membrane-targeted EGFP (EGFP-caax) which is separated from DeAct-GS1 by a self-cleaving P2A sequence (Fig. 4a). We first confirmed that expression of pMBP-DeAct-GS1 induced actin disassembly in cultured oligodendrocytes, while a control construct (pMBP-EGFP-caax alone) did not (Supplementary Fig. 6a–c).

To test whether inducing actin disassembly in early oligodendrocytes is sufficient to cause outfoldings, we targeted developing myelin in the mouse spinal cord. Compared to the inaccessible optic nerve, the dorsal spinal cord of neonatal mice is a tractable location for AAV injection and analysis of myelin ultrastructure^15,20^. A single injection of pMBP-EGFP-caax AAV at P0 was sufficient to achieve bright, widespread EGFP expression in oligodendrocytes throughout the entire spinal cord by P8 and is specifically enriched in the white matter (Fig. 4b and Supplementary Fig. 6d). Consistent with our prediction, inducing oligodendrocyte actin disassembly with DeAct-GS1 caused a 1.75-fold increase in myelin outfoldings (compared to EGFP-caax control), closely phenocopying OL-CalEx (Fig. 4c, d). Restricting DeAct-GS1 to the oligodendrocyte plasma membrane by direct fusion to a farnesyl-tag (DeAct-GS1-caax) also increased outfoldings (~2.9 fold increase; Fig. 4e, f), suggesting that cortical actin—actin closely associated with the plasma membrane^61,62^—may normally limit oligodendrocyte membrane expansion to prevent outfoldings (see Discussion).

Is actin filament assembly also required for myelin sheaths to achieve normal lengths? We first used myelinating cocultures^20,63^ to visualize and perturb actin filaments during the time of active sheath elongation along axons (Supplementary Fig. 7). Live-imaging of a genetically-encoded actin filament probe (pMBP-Ratiometric-Lifeact; see Methods) expressed in oligodendrocytes confirmed the presence of actin filaments at sheath edges during their elongation (Supplementary Fig. 7a–c). To test whether these actin filaments are required for myelin sheath elongation, we treated cocultures with the cell-permeable drug latrunculin to induce actin disassembly during early stages of myelination when sheaths are actively elongating along axons (Supplementary Fig. 7d). Inducing actin disassembly blocked further sheath elongation without causing sheath retraction or shortening (Supplementary Fig. 7e, f). Taken together, these coculture results suggest that a population of actin filaments at sheath edges may be required to promote sheath elongation along axons. Finally, to test this idea in vivo, we sparsely injected DeAct-GS1 (or EGFP-caax control) into the developing spinal cord and visualized individual myelin internodes (Fig. 4g). DeAct-GS1-expressing myelin sheaths were on average 21% shorter than control-expressing sheaths (Fig. 4h), closely phenocopying the sheath length defect we observed in OL-CalEx mice above. Together, these data suggest that calcium regulates actin filaments in oligodendrocytes to control the morphology and length of myelin sheaths.

### Genetically stabilizing actin in oligodendrocytes rescues myelin morphology defects in OL-CalEx mice

Would the stabilization of actin filaments in oligodendrocytes be sufficient to rescue the myelin morphology defects seen in the OL-CalEx mice? Given that actin disassembly is required for myelin wrapping^19,20^, we sought to identify a genetically encoded tool that would subtly increase actin filament levels but that would not block myelin formation. We used primary cultured oligodendrocytes to screen several genetic tool prototypes for inducing actin stabilization in cells (Supplementary Fig. 8). Our initial attempts at genetically stabilizing actin—likely too potent to be useful here—caused a pronounced increase in actin filament levels and dramatic cell morphology defects in cultured oligodendrocytes, closely resembling oligodendrocytes lacking the actin disassembly factors ADF (destrin) and cofilin (Supplementary Fig. 8a)^19^.

**Fig. 5:**
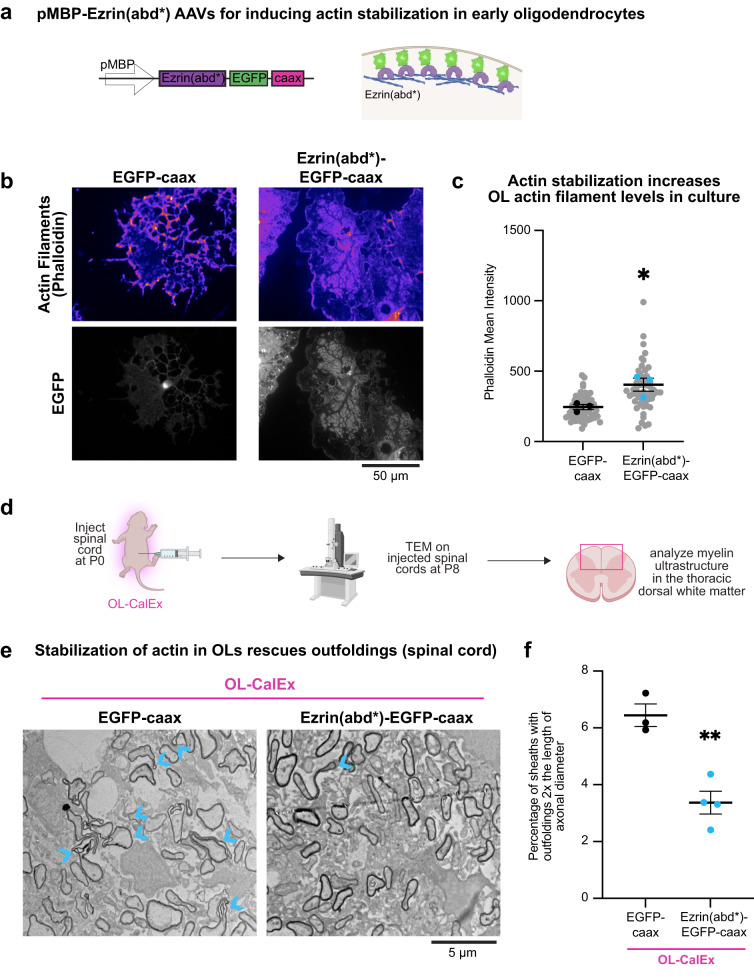
Genetically inducing actin stabilization in CalEx oligodendrocytes rescues myelin outfoldings. **a** Construct design to stabilize membrane-proximal actin in developing myelin sheaths: myelin basic promoter (pMBP), followed by constitutively-active Ezrin actin binding domain (Ezrin(abd*)) fused to EGFP-caax. Created with Biorender.com. **b** Epifluorescence representative micrograph of EGFP-caax (left) and Ezrin(abd*)-EGFP-caax (right) transfected oligodendrocytes stained with phalloidin (purple) to label actin filaments (top row) and endogenous EGFP expression (gray, bottom row). Scale bar, 50 μm. **c** Quantification of phalloidin mean intensity rat oligodendrocytes in **b**. Average ± SEM, *N* = 3 biological replicates (preps/bolded dots). Statistical significance determined by unpaired, two-tailed Student’s *t* test; *p = 0.0326. **d** Virus encoding for Ezrin(abd*)-EGFP-caax or control EGFP-caax were injected into P0 mouse pups. Injected spinal cords were harvested at P8 and processed for transmission electron microscopy. Created with Biorender.com. **e** Transmission Electron Microscopy of EGFP-caax injected (left) and Ezrin(abd*)-EGFP-caax (right) spinal cord sections. Scale bar, 5 μm. **f** Quantification of percentage of myelin sheaths with observed outfoldings twice the diameter of the axon in **e**. Average ± SEM, *N* = 3 EGFP-caax; *N* = 4 Ezrin(abd*)-EGFP-caax. Statistical significance determined by unpaired, two-tailed Student’s *t* test; **p = 0.0031.

Instead, to more subtly increase actin filament levels in oligodendrocytes, we turned to a construct that has been previously used in other cell types to induce stabilization of cortical actin without causing overt changes to cell morphology^61^—the constitutively-active actin binding domain of the cortical actin-binding protein Ezrin (Ezrin(abd*)-EGFP-caax; Fig. 5a). We engineered Ezrin(abd*)-EGFP-caax for oligodendrocyte-specific expression in vivo using the MBP promoter and packing in adeno-associated virus (AAV)^15,60^. We first confirmed that the expression of the Ezrin(abd*)-EGFP-caax increased actin filament levels in cultured oligodendrocytes compared to an EGFP-caax control, without grossly perturbing oligodendrocyte morphology (Fig. 5b, c). Next, to test whether Ezrin(abd*)-EGFP-caax perturbs myelin formation in vivo, we injected AAVs encoding for either MBP promoter-driven EGFP-caax or Ezrin(abd*)-EGFP-caax into WT pups. Inducing subtle actin stabilization in oligodendrocytes in vivo did not grossly affect myelin thickness or axonal caliber (Supplementary Fig. 8b, c), indicating that Ezrin(abd*) is optimally tuned to mildly stabilize actin filaments in oligodendrocytes without preventing myelination.

To test whether stabilization of actin filaments in oligodendrocytes is sufficient to rescue the outfolding defect in OL-CalEx mice, we injected AAVs encoding for either MBP promoter-driven EGFP-caax or Ezrin(abd*)-EGFP-caax into OL-CalEx pups (Fig. 5d). Consistent with the prediction that stabilizing actin filaments in developing myelin sheaths would rescue the outfolding defect, inducing actin stabilization in oligodendrocytes caused a 1.9-fold reduction in outfoldings, indicating that actin filament stabilization is partially capable of reversing sheath defects caused by calcium attenuation (Fig. 5e, f).

**Fig. 6:**
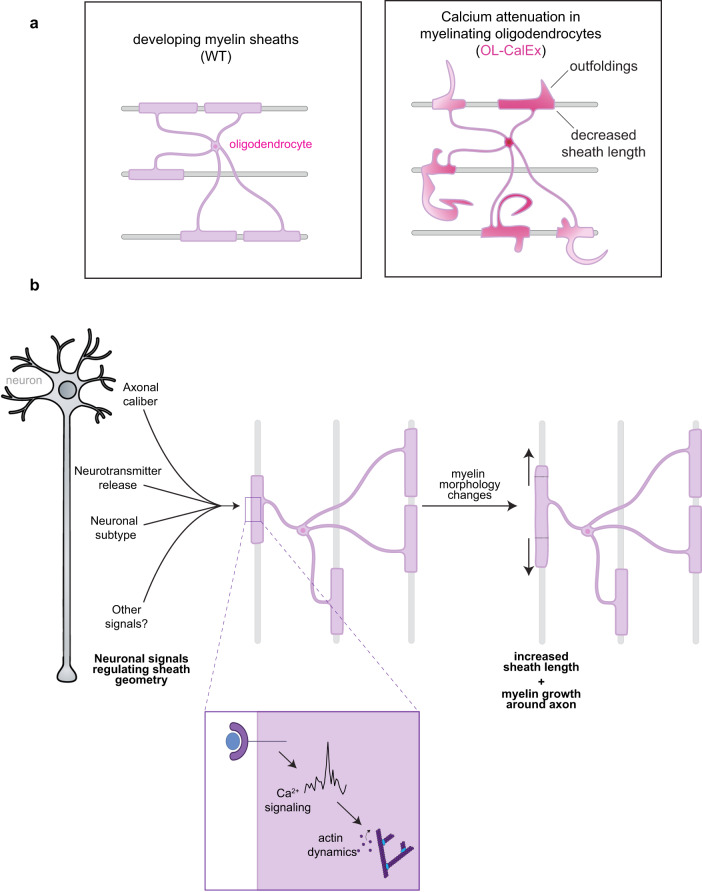
Model figure for oligodendrocyte calcium regulation of myelin sheath morphology. **a** (left) In developing myelin sheaths, calcium regulates actin filaments, which are required to guide myelin membrane accurately around neuronal axons. (right) Loss of calcium signaling leads to myelin outfoldings and shorter myelin sheaths, and both phenotypes are recapitulated by inducing actin disassembly in oligodendrocytes. Created with Biorender.com. **b** Model for how different neuronal properties (e.g. caliber, activity, etc.) may regulate myelin formation or dynamics by converging on oligodendrocyte calcium signaling. Inset shows oligodendrocyte calcium signaling promoting actin dynamics that are, in turn, required for normal sheath length and morphology. Created with Biorender.com.

Together, these results indicate that oligodendrocyte calcium signaling is required for actin-dependent regulation of myelin membrane morphology—and raise the possibility that this mechanism may allow myelin sheaths to respond to neuronal properties to fine-tune conduction velocity (Fig. 6).

## Discussion

How myelin sheaths are precisely sculpted during development to fine-tune nerve conduction velocity is a fundamental question in neurobiology. Prior studies suggest that calcium signaling in myelin sheaths is a potential mechanism that could allow oligodendrocytes to shape their myelin sheaths in response to the demands of the neurons they myelinate^34–36^. Here, we tested this idea by genetically attenuating calcium signaling in newly-formed oligodendrocytes in the developing mouse CNS using genetic tools including the calcium pump CalEx. We found that calcium signaling is dispensable for the differentiation and maturation of oligodendrocytes and for the initiation of myelination. However, attenuation of oligodendrocyte calcium signaling leads to shorter myelin sheaths and long outfoldings, suggesting that the major role of local calcium signaling in myelin sheaths is to properly sculpt sheath morphology. Mechanistically, we found that calcium signaling promotes actin cytoskeletal assembly in nascent sheaths. Accordingly, genetically inducing actin disassembly phenocopied the loss of calcium signaling in oligodendrocytes, while stabilization of actin filaments partially rescued the myelin outfolding defect in CalEx mice. Thus, a major role of calcium signaling in oligodendrocytes during developmental myelination is actin-dependent regulation of myelin morphology.

Our findings raise several questions. First, how does loss of calcium signaling cause myelin outfoldings? One possibility is that the outfoldings seen in OL-CalEx mice are a direct consequence of having shorter sheaths—the same amount of myelin membrane extending out into the neuropil rather than stretching along the axon (by analogy, outfoldings similar to the folds of a scrunched-down sock). If true, the pathological presence of outfoldings observed in other myelin mutants and neuropathies^11–14,17,20,64,65^ may also be a consequence of improper sheath extension. It will thus be interesting to determine whether other myelin mutants with short sheaths (e.g. Pak1, HCN2^66,67^) result in outfoldings. Alternatively, it is possible that sheath length defects are instead a downstream consequence of outfoldings. A third possibility is that outfolding formation occurs independent of sheath length, perhaps due to perturbed cortical actin filaments. In other cell types, cortical actin—actin closely associated with the plasma membrane—limits membrane expansion^61,62^. Our experiments using membrane-tethered tools to induce actin filament disassembly (Fig. 4) or stabilize actin filaments (Fig. 5) at the plasma membrane suggest that a membrane-proximal cortical actin cytoskeleton could locally restrict aberrant myelin membrane extension away from sheaths. This model predicts that regulated, local loss of the actin cytoskeleton—e.g. in the oligodendrocyte “inner tongue” (the leading edge that grows around the axon during wrapping)—could allow for myelin growth to be specifically confined to spirally wrapping around the axon, rather than away from the axon as in an outfolding. Whether newly differentiated oligodendrocytes have membrane-adjacent actin subpopulations and the role that these actin populations play in regulating myelin membrane growth remain open questions.

As a final potential explanation for the cause of outfoldings, it is possible that attenuating oligodendrocyte calcium signaling perturbs a normal developmental process of outfolding resolution, e.g. by inhibiting microglial pruning^17,68,69^. The reduction of outfoldings we observed from P21 to P60 in OL-CalEx mice (Fig. 2b, c) may represent such a developmental correction over time or simply a dilution of outfoldings over time as sheaths grow longer. Interestingly, the frequency of long outfoldings (length >2x the diameter of the corresponding axon) we observed in WT optic nerves closely matches the outfolding frequency previously reported by Snaidero et al^17^ for these time points (~1% of sheaths with outfoldings at P21, <1% at P60). However, the frequency of outfoldings we calculated in OL-CalEx nerves (100% of sheaths having 1-2 outfoldings; Methods Table 1) is higher than that calculated for wildtype nerves when outfoldings are at their most abundant (only 65% of WT sheaths with outfoldings somewhere along their length at P10^17^), suggesting that outfoldings are generated at a higher rate when calcium signaling is attenuated. Future studies using longitudinal in vivo imaging of sheaths in OL-CalEx mice may help resolve this question.

**Table 1:** Calculation for estimated percent of OL-CalEx sheaths with outfoldings

L (estimated average sheath length in OL-CalEx, 70% length of 150 μm sheath lengths previously reported)	OCL (outfolding connection length, from 3DSEM volumes)	CP (connection percentage: estimated % of sheath connected to outfolding = OCL / L • 100	MF, multiplication factor (100%/CP)	Percentage of OL-CalEx sheaths with long outfoldings in a given section, Fig. 2c	Estimated percent of sheaths with long outfoldings in OL-CalEx
105 μm	2.9 μm	2.8%	35.7	4.2%	149.9%

How does oligodendrocyte calcium signaling regulate sheath morphology during development? Actin filament dynamics are critical for normal myelin formation in the CNS^19,20^. We previously found that distinct stages of myelination have opposite requirements for actin filaments: actin filament assembly is required for oligodendrocytes to extend their cellular processes to ensheath axons, but the subsequent stage of myelin wrapping requires actin filaments to disassemble^20^. Our findings using CalEx mice agree with and build from this earlier work. Calcium signaling promotes actin filament assembly in nascent sheaths/early-stage oligodendrocytes but is not necessary for actin disassembly at later stages (Supplementary Fig. 5). Accordingly, myelin wrapping—which requires actin disassembly—is unaffected by calcium attenuation (Fig. 1). In contrast to wrapping, we find that the longitudinal elongation of myelin sheaths along axons requires calcium/actin signaling (Fig. 2g, h, Fig. 4g, h, and Supplementary Fig. 7). The magnitude of sheath length reductions we observed in OL-CalEx mice and mice expressing DeAct-GS1 are similar to sheath length reductions reported in the literature in the context of pharmacological/genetic perturbations^15,66,67^ as well as during motor learning^3^ (Supplementary Table 1). These findings raise the intriguing possibility that different aspects of myelin sheath morphology may be differentially regulated to precisely tune conduction velocity, i.e. calcium- and actin-dependent mechanisms controlling sheath length versus calcium/actin-independent control of myelin wrapping/thickness.

What are the molecular mechanisms that control calcium-mediated actin assembly in oligodendrocytes? One possible mechanism is via gelsolin, an actin disassembly factor that is highly expressed by oligodendrocytes^20,70^ and directly activated by calcium^71^. Inconsistent with gelsolin mediating the effect of calcium on actin regulation in oligodendrocytes, gelsolin knockout mice show no evidence of outfoldings (Supplementary Fig. 9a–c)^20^ and the major role of gelsolin during myelination appears to be to induce actin disassembly to drive wrapping^20^. Therefore, it is unlikely that gelsolin directly mediates calcium’s control over actin assembly in early stages of myelination. A second model for how calcium may regulate actin in oligodendrocytes is via its established role in promoting actin assembly through phosphoinositide (PI_4,5_P_2_) signaling and the N-Wasp-Arp2/3 axis^17^. Prior work^20,55,64,65^ and our findings here show that interfering with any step in this signaling pathway causes myelin outfoldings (Supplementary Fig. 9d). Moreover, loss of septins or anillin—an actin-binding protein required for septin assembly—are also sufficient for outfolding formation^72,73^. Together, these results converge on a model for myelin outfolding formation that may be shared by many disease states and myelin mutants—dysregulated actin assembly. It will be interesting to test this model in future studies.

What other cell biological processes does calcium regulate in oligodendrocytes? Recently, we discovered that myelin membrane and adhesion proteins are added via SNARE-mediated exocytosis^15^, a process that is well-known to be regulated by calcium in many other cell types^25,74,75^. Future studies will focus on whether oligodendrocyte exocytosis is regulated by calcium, and how actin and exocytosis may collaborate^76^ to regulate the morphology of myelin sheaths. In addition, it will be interesting to test whether calpains—the calcium-regulated proteases found to regulate myelin sheath lengths in developing zebrafish^34^—contribute to calcium-mediated sheath morphology in the developing or adult mouse CNS. If so, calpains may serve as a tool for determining the role of myelin sheath tuning in nervous system development or dynamics.

In contrast to the importance of calcium signaling during myelin formation, dysregulation of oligodendrocyte calcium signaling may be pathogenic in disease states^32^. Forced calcium influx into mature sheaths is sufficient to cause myelin decompaction and breakdown, a form of myelin pathology seen in response to numerous demyelinating insults^77–80^. Calcium has also been implicated in retraction of myelin paranodes as a consequence of excitotoxicity^81,82^. However, in many of these studies it remains unclear whether the primary driver of myelin pathology is calcium influx into neurons versus oligodendrocytes—a question that could be addressed with genetic tools like CalEx. More broadly, it is possible that the vulnerability of CNS myelin sheaths that causes myelin instability in disease and aging could be a consequence of the dynamic nature of these sheaths and ability to remodel in response to neuronal activity. Thus, it would be interesting to test whether CalEx or SpiCee-mediated calcium attenuation is protective in mouse models of demyelinating disease.

Why are OL-CalEx mice significantly smaller than their WT counterparts (Supplementary Fig. 1a, b)? One possible explanation is recombination in other cell types in the CNP-Cre. Loss of calcium signaling in these cell types (including Schwann cells or a small population of spinal cord neurons) could lead to small overall body size^83–85^. Due to these limitations of the CNP-Cre, we developed fully-orthogonal pMBP-driven AAV tools (including SpiCee, DeActs, and Ezrin(abd*)). Since pMBP-driven SpiCee and DeActs phenocopy OL-CalEx mice (Fig. 4 and Supplementary Fig. 4) and pMBP-Ezrin(abd*) partially rescues outfoldings, we conclude that the myelin sheath defects we observed in OL-CalEx mice are due to calcium/actin perturbations within oligodendrocytes, rather than an indirect consequence of CalEx expression outside of the oligodendrocyte lineage.

What is the physiological role of calcium-regulated myelin sheath morphology in the developing nervous system? Oligodendrocyte calcium signaling may be a mechanism by which an oligodendrocyte can adjust its myelination patterns based on properties of the neuron that it is myelinating. Previous work has shown that myelin sheath length and thickness are dictated by neuronal properties including axonal caliber, neuronal type, and neuronal activity^10^. However, how myelin senses and responds to neuronal properties to build sheaths of the proper length and thickness remain unknown. Calcium influx into the cytosol can be regulated by a diverse set of mechanisms including voltage gated and mechanosensitive channels, store-operated channels and release from the endoplasmic reticulum, mitochondrial permeability pore opening, sodium/calcium exchangers, gap junctions, and neurotransmitter receptors—all of which may regulate calcium signaling in oligodendrocytes in response to the properties of the neurons being myelinated. For example, calcium signaling in oligodendrocytes can be modulated by neuronal activity in zebrafish^35^, and can be triggered by neurotransmitters including ATP and glutamate—at least in cultured oligodendrocytes^32,33^. Oligodendrocyte calcium signaling is thus poised to act as an essential cell-signaling bridge between these various neuronal properties and myelin.

Does oligodendrocyte calcium signaling also drive myelin remodeling to support learning in the adult CNS? Experience and neuronal activity cause dramatic changes to the pattern of myelin in the adult brain, including addition of new myelin sheaths and morphological changes to existing sheaths^3–9^. Although such changes are predicted to affect the function of neuronal circuits, the overarching role of myelin dynamics in learning is still mostly hypothetical. An essential next step for the field is to determine the cellular mechanisms that drive activity-induced myelin remodeling, and then leverage that knowledge to be able to test the importance of myelin remodeling across learning modalities. Could oligodendrocyte calcium signaling be the missing mechanistic link between neuronal activity and plasticity-induced changes to myelin? While the precise role of neuronal activity in driving oligodendrocyte calcium signaling remains an open question^36,37^, several lines of evidence converge to support the idea that neuronal activity could regulate oligodendrocyte sheath morphology via calcium signaling. First, blocking neuronal activity or neurotransmitter release perturbs myelin sheath lengths or sheath number per oligodendrocyte in zebrafish^86–89^. Second, several neurotransmitters including glutamate and ATP are sufficient to elicit calcium transients in oligodendrocytes, although the extent to which this effect persists as oligodendrocytes mature remains to be elucidated^32,33^. Third, neuronal activity promotes sheath elongation^86^ and calcium transients in sheaths^35^. Together, these results suggest that local calcium transients in sheaths could respond to neuronal activity to control sheath remodeling. Although these findings were specific to development, similar mechanisms could potentially occur during learning-induced oligodendrogenesis or sheath remodeling in the adult. For example, it is possible that neuronal activity-induced calcium signaling in oligodendrocytes directly regulates actin dynamics to power plasticity-induced myelin remodeling, similar to calcium’s role in nascent sheaths during development that we uncovered here.

In summary, we discovered that calcium signaling is required for the actin-dependent sculpting of myelin sheath morphology during development. We propose that this mechanism could allow myelin sheaths to tune conduction velocity in response to diverse neuronal signals, including activity. Our work provides a conceptual framework for future studies to understand how oligodendrocyte calcium signaling contributes to myelin remodeling during learning and myelin loss/regeneration in the context of disease.

## Methods

### Animals

All procedures involving animals were approved by the Institutional Administrative Panel on Laboratory Animal Care (APLAC) of Stanford University and followed the National Institutes of Health guidelines under animal protocol APLAC 32260. Animals were group-housed in a Stanford University animal facility with a 12:12 h light/dark cycle. Mouse rooms were kept between 18-23 degrees Celcius and 40-60% humidity. Mice were given ad libitum access to food and water. Mice were housed in plastic cages with disposable bedding. All mice were monitored by veterinary and animal care staff. Animals used in the study had not undergone prior procedures. CalEx fl/fl (which contain a floxed STOP cassette in front of CalEx-mCherry) mice were gifted by Drs. Xinzhu Yu and Baljit Khakh. Cnp-CRE/+ mice were obtained from Dr. Klaus Nave (Max Planck Institute for Experimental Medicine) and maintained through crossing to C57Bl/6 mice. The OL-CalEx line was generated by crossing homozygote CalEx fl/fl with heterozygote CNP-Cre/+ mice. Sprague-Dawley rats and C57BL/6 mice were ordered from Charles River Laboratories. The following primers were used for genotyping: Common CalEx forward primer 5’– CCTTTCTGGGAGTTCTCTGCTGC–3’; WT Reverse CalEx 5’– GCGGATCACAAGCAATAATAACCTG–3’

Mutant Reverse CalEx primer 5’– CGTAAGTTATGTAACGCGGAACTCC–3’. CNP forward primer 5’—GCCTTCAAACTGTCCATCTC—3’; CNP reverse

5’—CACCATTATTTTCCCGACCC—3’. All resulting progeny from these crosses were used; CalEx/+; CNP-Cre/+ mice are referred to as “OL-CalEx.” Sprague-Dawley rats and C57BL/6 mice were ordered from Charles River Laboratories. Male and female mice were used for all in vivo experiments. For cell culture studies, brains of both sexes were pooled to obtain sufficient cell numbers.

For all histology, animals were anesthetized with injecting a cocktail of ketamine (100 mg/kg) and xylazine (20 mg/kg). Animals were then transcardially perfused with PBS and tissues were dissected and drop fixed with 4% paraformaldehyde (PFA) (made from 16% PFA, Electron Microscopy Sciences) for immunohistochemistry or Karlsson and Schultz (KS) fix for transmission electron microscopy.

### Isolation of oligodendrocyte precursor cells

Primary OPCs were purified by immunopanning from P5-P7 Sprague Dawley rat or P6-P8 transgenic mouse brains as previously described^90,91^. Briefly, rat cortices were digested with papain enzyme and were serially passed over plates to negatively select for astrocytes (Ran2), mature oligodendrocytes (GalC), before finally positively selecting for OPCs. OPCs were typically seeded at a density of 50,000-250,000 cells/10-cm dish and recovered for 4 days in culture before lifting cells via trypsinization and distributing for transfection, proliferation, or differentiation assays. All plasticware for culturing OPCs were coated with 0.01 mg/ml poly-D-lysine hydrobromide (PDL, Sigma P6407) resuspended in water. All glass coverslips for culturing OPCs were coated with 0.01 mg/ml PDL, which was first resuspended at 100x in 150 mM boric acid pH 8.4 (PDL borate).

To proliferate primary OPCs, cells were cultured in a serum-free defined media (or “DMEM-SATO” base media) supplemented with 4.2 μg/ml forskolin (Sigma-Aldrich,Cat#F6886), 10 ng/ml PDGF (Peprotech, Cat#100-13 A), 10 ng/ml CNTF (Peprotech, Cat#450-02), and 1 ng/ml neurotrophin-3 (NT-3; Peprotech, Cat#450-03) and house in an incubator set to 37 C with 10% CO2. To induce differentiation, cells were switched to DMEM-SATO base media containing 4.2 μg/ml forskolin (Sigma-Aldrich, Cat#F6886), 10 ng/ml CNTF (Peprotech, Cat#450-02), 40 ng/ml thyroid hormone (T3; Sigma-Aldrich, Cat#T6397), and 1x NS21-MAX (R&D Systems AR008).

### Plating schemes and pharmacological cell treatments

Primary OPCs from CalEx/+;Cnp-Cre/+ and control littermate mice were harvested and cultured as described above in Purification and Culturing of Cells. Cells were seeded onto 12-mm glass coverslips (Carolina Biological Supply No. 63-3029) at a density of 5,000 cells/coverslip in differentiation media. Cells were cultured until day three in differentiation and then fixed for subsequent staining analysis.

For all BAPTA-AM (ThermoFisher B6769) or dimethyl BAPTA-AM (DMB-AM; Sigma-Aldrich 16609-10MG-F) experiments, wildtype rat OPCs were plated in differentiation media at a density of 10,000 cells/coverslip. Briefly, BAPTA-AM or dimethyl-BAPTA-AM stocks were resuspended in tissue culture grade DMSO at a concentration of 1 μM. Drugs were then diluted to 2x final concentration in completed DMEM-SATO with differentiation factors. 250 uL of media was removed from each well and replaced with 250 μL of drug or DMSO-containing media. Final drug concentrations were 1μM or 500 nM. Previous reports have found that at high micromolar concentrations (50 μM), BAPTA can cause calcium-independent disassembly of the actin and microtubule cytoskeletons in cultured cells, but that variants of BAPTA such as dimethyl-BAPTA do not have these off-target effects on the cytoskeleton^38^.

### Myelinating co-cultures with retinal ganglion cells

Our protocol for myelinating co-cultures with CNS-derived axons was adapted from previous publications^63,92^ with minor modifications. Dissection and dissociation of retina, immunopanning for retinal ganglion cells (RGCs) with anti-Thy 1.1, and media composition with growth factor supplementation was performed as previously published.

To create re-aggregates, we seeded freshly harvested RGCs into UV-sterilized PCR tubes (Fisherbrand 14-230-215) at a density of 10,000 cells/100 μ l of media. After 24 h of recovery, we half-fed each tube by exchanging 50 μ l of media with freshly supplemented growth factors. After 48 h of recovery, we transferred the 100 μ l-cell suspension containing re-aggregated clumps of RGCs from each tube onto a PDLborate coated 12 mm glass coverslip situated in a 24-well plate (or onto 8-well chamber dishes for live imaging, Ibidi 80827). Each well was half-fed with fresh growth factors every 72–96 h. After 10–14 days, the RGC re-aggregates formed dense beds of radially protruding axons.

Rat oligodendrocyte precursors freshly harvested from P5 to P7 brains were seeded directly onto RGCs at a density of 40,000 cells/well. For experiments involving AAV-infections of oligodendrocytes, appropriate amount of AAV (~2 μL of 1 × 10^11^ vg/mL titer pMBP-drive AAV per 40 K oligodendrocytes) was added to media containing oligodendrocytes, which were then seeded directly onto the RGCs. After 24 h, 1 μm of the gamma-secretase inhibitor DAPT (Calbiochem Cat. No. 565784) was added to each well to promote ensheathment.

### Latrunculin-A treatment of co-cultures

Latrunculin-A (LatA, InvitrogenL12370) stocks were resuspended in tissue culture grade DMSO at a concentration of 1 mM. Drugs were then diluted to 10x final concentration in co-culture media. At the indicated time point, 50 uL of media was removed from each well and replaced with 50 uL of drug or DMSO containing media. Final drug concentrations were 500 nM on the first day of treatment, and 125 nM on each subsequent treatment day (see timeline in Supplementary Fig 7a). All co-cultures were fixed after a total of 6 days in culture for immunofluorescence as previously described in published protocols^63,92^. Briefly, cells were incubated in 4% PFA at room temperature for 15 minutes, washed 3 times post-fix, and stored in at 4 degrees until staining.

### Staining and Imaging of Latrunculin-A treated co-cultures

Fixed co-cultures were stained with rat anti-MBP (Abcam ab7349, 1:500) and mouse anti-NF200 (Sigma N0142, 1:100), followed by donkey anti-rat Alexa Fluor 594 (Thermo Scientific A-21209, 1:1000) and donkey anti-mouse Alexa Fluor 594 (Thermo Scientific A-21203, 1:500). Stained cells were mounted onto microscope slides in Fluoromount G with DAPI to label nuclei.

Cells were imaged by widefield epifluorescence with a Zeiss Axio Observer Z1 using the Plan-Apo 10x objective. Images were acquired and analyzed blinded to condition with identical illumination and acquisition conditions per biological replicate. MBP+ area per image was quantified in ImageJ by deleting regions with background fluorescence from RGC aggregates, then using a consistent threshold across images to measure total MBP+ area per image. Total number of MBP+ oligodendrocytes was then quantified using the Cell Counter function, and the reported MBP+area/cell value was generated by dividing the total MBP+ area by the number of MBP+ oligodendrocytes. We used this metric instead of directly measuring sheath length because the densely packed RGC axons lead to oligodendrocytes sheaths that cross over each other, making individual MBP+ sheaths difficult to reliably measure.

### Design of DeAct-GS1-EGFP-caax, Ezrin(abd*)-EGFP-caax, and Ratiometric Lifeact constructs

DeAct-GS1—EGFP-caax and Ezrin(abd*) constructs were created using InFusion cloning (Takara Bio) by annealing one DNA fragment into the parent plasmid. The parent plasmid contains a 1.3 kb MBP promoter driving expression of (farnesylated, membrane-bound) EGFP-caax in a pAAV vector backbone and was linearized using NcoI and BglI. The fragment encoding for either human gelsolin segment 1 (GS1, the actin-severing domain; aa 53-176, Addgene # 89445)^59^ or human Ezrin(abd*) (Ezrin aa 552-586 with T567D constitutively-active mutation, Addgene #155227)^61^ was cloned with 15 base pair overhangs required for InFusion reactions. This created constructs with the following configuration: pMBP-GS1-EGFP-caax or pMBP-Ezrin(ABD*)-EGFP-caax. pMBP-EGFP-caax AAV vector (Addgene #190155) was previously reported^15^.

To detect actin filaments in live cells, genetically encodable Lifeact^93^ is the field standard. However, Lifeact has been shown to bind actin monomers. To overcome this issue and visualize only actin filaments, we generated Ratiometric Lifeact (RMLA). RMLA constructs were created using InFusion cloning (Takara Bio) by annealing two DNA fragments into the parent plasmid. The parent plasmid contains a 1.3 kb MBP promoter driving expression of Lifeact-mRuby3 in a pAAV vector and was linearized using BamHI and BglI. Two fragments encoding self-cleaving peptide P2A-T2A^94^ and mClover3 (cytoplasmic) were PCR-amplified with 15 base pair overhangs required for InFusion reactions and inserted into the backbone. This created constructs with the following configuration: pMBP-Lifeact-mRuby3-P2AT2A-mClover3. This (in principle) results in equimolar production of Lifeact-mRuby3 and cytoplasmic mClover3 in a cell, which can be divided to generate a ratiometric signal specific for actin filaments. All DNA constructs generated for this paper will be made publicly available by AddGene at the time of publication. All correspondence and requests for materials should be addressed to J.B.Z.

### Transfection of OPCs

Rat OPCs were trypsinized and dissociated from tissue culture dishes and centrifuged at 90 × *g* for 10 min. 250,000 OPCs were gently resuspended in 20 μl of nucleofector solution (Lonza P3 Primary Cell 4D-Nucleofector V4XP-3032) with 400 ng of EGFP-Caax, GS1-P2a-EGFP-Caax, or GS1-Caax plasmids. Cells were loaded into a 16-well cuvette and electroporated in a Lonza 4D-Nucleofector X Unit (AAF-1003X) assembled with a 4D-Nucleofector Core Unit (AAF-1002B) using pulse code DC-218. Electroporated cells rested for 10 min at room temperature. 80 μl of antibiotic free DMEM-SATO media was added to each cuvette and cells were gently triturated. Each well of 250k cells was distributed onto No. 1 glass coverslips coated with PDL-borate for differentiation timepoints and technical replicates (up to six coverslips from a single transfection). Each coverslip was half-fed with freshly supplemented DMEM-SATO media every two to three days.

### Calcium imaging

Immunopanned CalEx; Cnp-Cre OPCs were differentiated for two to three days for all calcium imaging experiments. To load 1 μM cell permeable calcium indicator, Fluo-4-AM (Invitrogen, F14201) 50 μg tube of lyophilized Fluo-4-AM stock was resuspended in 46 uL of sterile DMSO (1 mM concentration). 1.5 μL of Fluo-4-AM stock was added directly to cell media to prevent shearing of cells. Cells were incubated with Fluo-4-AM for 20 minutes and then media was completely changed to 1 mL DMEM-SATO Media (made with Fisher Scientific A1896701) for imaging.

Cells were imaged on an Opterra II Multipoint Swept Field Confocal outfitted with a humidified, temperature-controlled microscope enclosure (Okolab microscope enclosure, H201-Temperature Unit, CO2 controller, HM-Active Vibration Free Humidity Controller with humidity sensor and temperature-controlled tube). Imaging was performed using the 60x/1.2 NA water objective and Perfect Focus to prevent z-plane drift during imaging. Images were acquired every two seconds in 488 channel with 70 μM slit and 100 ms exposure time with 15% laser power. Calcium imaging data was analyzed using Fiji to select regions of interest and was analyzed to extract rate and amplitude measurements. All calcium imaging was performed on oligodendrocyte processes; soma events were relatively rare compared to process events. Data was analyzed blind to condition.

### Immunostaining of primary oligodendrocytes

At the specified day of differentiation, cell media was removed, and coverslips were treated with 4% PFA for 15 min at RT, followed by three washes with 1xPBS and permeabilization in 0.1% Triton X-100 in PBS for 3 min at RT. Prior to staining, cells were incubated in a blocking solution of 3% BSA in PBS for 20 min at RT. Then, primary antibodies (rat anti-MBP and/or rabbit anti-RFP) were added in a 3% BSA solution for overnight incubation at 4 C. On the following day, the primary antibody solution was rinsed off with three washes of PBS, and then incubated with secondary antibodies (anti-rat AlexaFluor 594 or 647) in 3% BSA for 1 hr at RT. After three washes with PBS, CellMask Blue stain (1:1000) and phalloidin-488 (7 μL phalloidin per 1 mL PBS) was incubated to stain all cells for 15 min at RT, followed by three additional rounds of washing with PBS. Stained cells were mounted onto microscope slides (Fisher Scientific 12-550-143) in Fluoromount G (SouthernBioTech, 0100-20).

Cells were imaged by widefield epifluorescence with a Zeiss Axio Observer Z1 using the Plan-Apo 20x/0.8 NA objective for actin, MBP, and cell area quantifications. Images were acquired blinded to the genotype or condition with identical illumination and acquisition conditions per biological replicate.

### Live-cell imaging of Ratiometric Lifeact in myelinating co-cultures

Time-lapse imaging was performed on a Zeiss Axio Observer Z1 inverted microscope equipped with a Zeiss Axiocam 506 monochrome 6-megapixel camera, a stage top incubator (Okolab, H301-K-Frame) set to 37 °C, and a digital gas blender (Okolab, CO2UNIT-3L) set to 10% CO 2 during image acquisition. Samples were imaged with a Plan-Apo 20X air objective using widefield epifluorescence with a 12 V halogen lamp. Time-lapse sequences of mClover3 (488) and Lifeact-mRuby3 (594) were acquired for 20 cells every 2 h for 14 h using Zen Blue software. Low frame rate (2hrs) was used to avoid phototoxicity during long-term imaging. One image in a region away from cells was also imaged to be used for background subtraction to correct for uneven illumination.

### Processing Ratiometric Lifeact signal

To produce RMLA images showing actin filaments, mClover3 and Lifeact-mRuby3 images were background subtracted by using Fiji’s “Image Calculator” function to subtract the background image acquired away from cells from the images. Each mClover3 and Lifeact-mRuby3 frame was then aligned using Fiji’s “Template Matching” plugin (channel alignment is crucial for ratiometric images). A ratiometric image was then made following previously published methods^61^. Briefly, Fiji’s “Image Calculator” function was used to divide the Lifeact-mRuby3 signal by mClover3, producing a 32-bit Ratiometric Lifeact image (see Supplementary Fig. 7b for examples of each channel and resulting RMLA image). This resulting image was filtered with a 1-pixel median filter to remove single-pixel noise and viewed with the Fire LUT.

### Spinal cord injections

AAV-DJ serotype virus was produced by the Gene Vector Virus Core at Stanford University and stored at −80 °C until use in experiments. Virus was thawed and diluted 1:1 with Trypan Blue for high-titer injections or 1:8 (for 1 μL of virus, 1 μL of Trypan Blue, and 6 μL of sterile D-PBS) for sparse labeling experiments. Viral titers were as follows:

**Table:** 

Virus Name	Genomic titer (viral genomes/mL)
AAV DJ pBZ442 pMBP-SpiCee-mRuby3	4.60E + 12
AAV DJ pBZ 423 pMBP-GS1-EGFP-caax	6.05E + 12
AAV DJ pBZ 424 pMBP-Ezrin(abd*)-EGFP-caax	2.17E + 13
AAV DJ pBZ 281 pMBP-EGFP-caax	1.48E + 13
AAV DJ pBZ 283 pMBP-GS1-P2A-EGFP-caax	2.75E + 13

P0-P1 C57/Bl6 mouse pups were placed in a KimWipe and deeply anesthetized on ice until unresponsive to a toe pinch. Using a Hamilton syringe (Model 80308 701SN, Point Style 4, 32 gauge, 20 mm length, and 12°), AAVs were injected into the lumbar spinal cord of mouse pups; successful injections lead to a blue stripe, indicating that virus had traveled throughout the spinal cord. Pups recovered at 34 °C on a heating pad until pink and moving freely and then were returned to the home cage. Spinal cords were then extracted as described (see Animals) at the appropriate age and processed either for immunohistochemistry (IHC) or TEM.

### Immunohistochemistry of spinal cords

Freshly dissected spinal cords were drop fixed in 4% paraformaldehyde for 5 h, washed with PBS, and then dropped into 30% sucrose overnight. Fixed spinal cords were sectioned on a cryostat (CM3050S, Leica Microsystems) into 30 μm sections (Olig2/CC1 staining) or 10 μm sections (SiR-actin/MBP staining), and immediately mounted onto Superfrost Plus (VWR) microscope slides. Slides were dried at 34 °C for 10 minutes to ensure section adherence, and then stored at −80 °C until subsequent use.

Prior to staining, slides were warmed at 34 °C for 30 minutes, sections were circled with a hydrophobic pen (ThermoScientific 008899) and dried for another 20 minutes. Sections were permeabilized with PBST (0.1% Triton X-100 in PBS) for 5 minutes at RT, blocked with 10% donkey serum in PBST for 1 h and then primary antibodies diluted in 1% donkey serum in PBST were applied (dilutions specified in Antibodies section) and incubated at 4 °C overnight. The next day, primary antibody was rinsed with 3 ×20 minute washes in PBS. Secondary antibodies and SiR-actin diluted in 1% donkey serum in PBST were incubated for 2 h at RT. Secondary antibody was washed 3 ×20 minutes in PBS. Samples were mounted in Vectashield with DAPI (Vector, H-1200) using a coverslip.

Stained spinal cord sections were imaged by Airyscan (super-resolution) confocal microscopy using a Zeiss LSM 880 laser scanning confocal microscope with a 63×/1.4 NA oil objective lens, using Zeiss Zen Blue software. Airyscan super-resolution imaging doubles the x-y resolution and improves signal-to-noise 8-fold compared to a regular confocal microscope and allows for gentle imaging to reduce photobleaching (ideal for quantitative fluorescence imaging)^95^. Photobleaching was further minimized by setting laser power to 1% and by focusing/setting levels on a region of tissue far from the actual regions imaged to preserve fluorescence of regions of interest for quantification.

For MBP/SiR-actin experiments, 5 biological replicates were used per group; from each biological replicate at least 2 spinal cord sections (technical replicates) were first averaged to calculate a mean for each animal. We previously found that averaging technical replicates was needed for accurate fluorescence measurements, likely due to mild variability in tissue section thickness, which can effect staining^20^. For analysis, all MBP+ rings were selected as ROIs, and mean intensity was measured in the SiR-actin channel. All the individual intensities across all the technical replicates from a single biological replicate were averaged together to calculate the mean intensity for a biological replicate. Statistics were performed on biological replicates. SiR-actin is a fluorogenic probe based on the specific and high-affinity actin filament-binding molecule jasplakinolide^56^. We confirmed specificity of SiR-actin for actin filaments in P20 spinal cord tissue sections by blocking staining with (unlabeled) jasplakinolide (Supplementary Fig. 10a, b), as we previously did for phalloidin staining^20^. We also imaged and quantified a dilution series of SiR-actin in PBS in custom-built flow chambers (parafilm with 0.5 cm diameter circular cutouts sandwiched between a glass slide and coverslip) using identical acquisition parameters as used for tissue sections, which show that this method results in linear, quantifiable differences in fluorescence intensities (Supplementary Fig. 10c). Reproducibility of this staining/imaging/ quantification methodology across technical (adjacent tissue sections) and biological (different mice) replicates is shown in Supplementary Fig. 10d.

### Antibodies and staining reagents

Primary antibodies used in this study were as follows: Rat-anti-MBP (Abcam ab7349; 1:100), Mouse-anti-CC1 (Millipore Sigma Ab-7, #OP80; specific for Quaking 7 protein enriched in oligodendrocytes^96^, 1:500), Goat-anti-Olig2 (R&D Systems AF2418; 1:500), Rb-anti-RFP (Rockland #600-401-379; 1:1000). Secondary antibodies used in this study were as follows at a 1:1000 dilution for both tissue and primary cell immunofluorescence: donkey anti-rat Alexa Fluor 594 (Thermo Scientific A-21209), goat anti-rat Alexa Fluor 647 (Thermo Scientific A-21247), donkey anti-mouse Alexa Fluor 488 (Thermo Scientific A-21202), donkey anti-mouse Alexa Fluor 647 (Thermo Scientific A-31571), donkey anti-rabbit Alexa Fluor 594 (Thermo Scientific, A- 21207) Alexa Fluor 488 conjugated Phalloidin (Thermo Scientfic, A12379), SiR Actin (Cytoskeleton, CY-SC001; 10 μM), HCS CellMask Blue (Thermo Scientific H32720; 1μg/mL) were also used where noted.

### Validation of antibodies

Rat-anti-MBP: We previously validated Abcam ab7349 for immunostaining mouse CNS tissues and primary oligodendrocytes using Shiverer (MBP-null) mice (Zuchero et al., 2015; PMID:26166300).

Mouse-anti-CC1: This antibody was validated to specifically recognize Quaking 7, a protein highly enriched in differentiated oligodendrocytes, and is commonly used to mark the cell bodies of differentiated oligodendrocytes (Bin et al., 2016; PMID:27454326).

Goat-anti-Olig2: Not validated to our knowledge, but cited in 290 publications and used for immunostaining (https://www.citeab.com/antibodies/688937-af2418-human-mouse-rat-olig2-antibody?des=848da71fda673fbf).

Rabbit-anti-RFP: We validated this antibody for immunostaining of mCherry by comparing CalEx-mCherry-expressing mice to littermate controls not expressing CalEx-mCherry (Fig. S1, c-d). Also cited in 1260 publications (https://www.citeab.com/antibodies/1908633-600-401-379-anti-rfp-rabbit-antibody-min-x-hu-ms-a?des=60601c11e690e5fc).

### Scanning electron microscopy sample preparation

Samples were processed and imaged as previously described^97^. Briefly, materials were sourced from Electron Microscopy Sciences (Hatfield, PA) unless otherwise stated. Optic nerves were dissected from euthanized animals and then immediately drop fixed in Karlsson-Schultz fixative (2.5% glutaraldehyde, 4% PFA in phosphate buffer, pH 7.3) until SEM processing. Fixed samples of optic nerve were carefully dissected and immersed in a modified Karnovsky’s fixative (4% paraformaldehyde, 2.5% glutaraldehyde, 0.1 M sodium cacodylate, 3 mM calcium chloride) for at least 24 h. Samples were rinsed repeatedly with ice-cold buffer (0.1 M sodium cacodylate, 3 mM calcium chloride) before further fixation with reduced osmium (1% osmium tetroxide, 1.5% potassium ferrocyanide, 0.1 M sodium cacodylate, 3 mM calcium chloride). Samples were rinsed repeatedly with ice-cold water and stained with 1% aqueous uranyl acetate overnight at 4 °C. The following day, samples were rinsed thoroughly with ice-cold water and serially dehydrated in ascending concentrations of ice-cold ethanol. Samples were finally rinsed in three changes of anhydrous ethanol at room temperature before infiltration with a 1:1 mixture of anhydrous ethanol and epoxy resin (Epon 812, hard formulation) for 4 h on a rotating mixer, followed by infiltration in pure resin overnight on a rotating mixer.

The following morning, samples were transferred into polypropylene bottlecaps with fresh resin and allowed to infiltrate for two hours before flat embedding in silicone molds, with nerves oriented to facilitate cross-sectioning. Blocks were polymerized at 70 °C for 48 h.

Ultra-thin sections were collected using a diamond knife (Diatome Histo 6 mm) and an ultramicrotome (Leica UC7). The blockface was carefully trimmed tight to the nerve in cross section using razor blades, and ultrathin sections (45-60 nm) were transferred using a wire loop onto diced chips of silicon wafer, and dried on a hotplate set to 60 °C. The silicon chips with adhered ultrathin sections were labeled with a diamond-tipped scribe and mounted on aluminum stubs using sticky carbon tabs before loading into the scanning electron microscope (Zeiss Sigma VP).

Images were collected using a backscattered electron detector (Gatan) at a working distance of ~6 mm, with accelerating voltage set to 3 kV, a 30 µm aperture, and the beam in high current mode. Atlas5 control software (FIBICS) was used to capture a low magnification view of each section, and 5–7 high-resolution fields (4 nm/px) of at least 20 µm per side sampled from across the nerve cross section that were used for analysis.

### 3D EM imaging and visualization

Imaging of serial sections by scanning EM (S3EM) was conducted as previously described^97,98^, with some modifications. Briefly, samples used in 2D analysis from control and CalEx conditions were selected and blockfaces were trimmed using a 90° diamond trimming knife (Diatome) to a trapezoidal frustum of roughly 150×400 µm. A silicon chip (35x7mm; University Wafer, Boston, MA) was hydrophilized in a plasma cleaner (Harrick), rinsed in pure water, and partially immersed in a Diatome Histo knife, with one end sticking out of the water at the back of the boat. Four drops of pure ethanol were added to the water in the boat to attenuate surface tension, and an ionizing gun (Leica EM Crion) was activated and oriented towards the cutting edge of the knife mounted on the ultramicrotome. Ribbons of approximately 100 serial sections of a nominal 55 nm thickness were cut. When ribbons of sufficient quality and length were generated, they were released from the knife edge using a single-eyelash brush and carefully positioned over the chip. The water level was then slowly lowered, and sections were allowed to dry down on the silicon substrate over a few minutes. Chips were further dried on a hot plate set to 60 °C for approximately 5 minutes and immediately labeled with a diamond scribe to indicate animal ID and nominal section thickness.

Samples were loaded into the same scanning EM microscope and imaged with the same imaging conditions using the array tomography software module of Atlas5. Briefly, low resolution (100 nm/px) image maps of the ribbon(s) of serial sections were generated, and a mid-resolution (50 nm/px) map of a central section was collected for evaluation. A region of interest (ROI) unobstructed by artifact throughout the series was identified and high-resolution (8 nm/px) images were collected from the ROI identified on consecutive sections. Following image intensity normalization in MIB2^99^, images were rigidly aligned using TrakEM2 in Fiji^100^, and fine alignment was accomplished using SWiFT-IR with compute resources provided from TACC through the 3dem.org portal^101^. The datasets for each animal constituted volumes of at least 15 × 15 × 4 µm in dimension (with voxel sizes of 8 × 8 × 55 nm).

Segmentation of myelin outfoldings and their corresponding axons was performed manually using the Volume Annotation and Segmentation Tool (VAST)^102^. The serial 2D segments were exported as.obj mesh files using the VAST Tools Matlab package. Meshes and data were imported into Blender software (blender.org) with the Neuromorph addon for further visualization^103^.

### TEM sample preparation and quantification of 2D electron microscopy

Transmission electron microscopy (TEM) was completed in the Stanford Cell Sciences Imaging Facility. Samples were prepared according to previously published protocols^104^. Samples were washed in cold Karlsson-Schultz fixative (2.5% glutaraldehyde, 4% PFA in phosphate buffer, pH 7.3) and incubated in 2% OsO4 for four hours at 4 °C with gentle shaking. The samples were then serially dehydrated in ethanol at 4 °C and embedded in EmBed812 (EMS, 14120). 80 nm sections were taken using an UC7 (Leica, Wetzlar, Germany) and were collected on formvar/Carbon coated 100 mesh Cu grids. Sections were stained for 40 seconds in 3.5% uranyl acetate in 50% acetone followed by staining in Sato’s lead citrate for 2 minutes. Sections imaged in the JEOL JEM-1400 120 kV. Images were taken using a Gatan OneView 4k X 4k digital camera. Quantification of TEM and SEM images was performed manually onusing Fiji/Image-J. For percentage of myelinated axon quantifications, axons were classified into one of three categories: (1) unmyelinated, (2) ensheathed (one to three loose wraps of myelin, or (3) myelinated (electron dense, compact myelin visible). For g-ratio analyses, we manually calculated axonal caliber and myelin thickness to calculate g-ratio. For outfolding analysis, we quantified the percentage of outfoldings seen in all myelinated axons in a field of view, and outfoldings of “extreme length” were defined as being twice the length of the diameter of the associated axon. Number of axons quantified per animal for percentage myelinated axons and g-ratio: 193–500. For outfolding quantifications: 632–1000 axons. All quantification were performed from 3 to 4 nonadjacent fields of view.

### Estimation of actual outfolding frequency from electron microscopy images

Electron microscopy can visualize only an ultrathin ( ~ 55-90 nm) section of tissue, but the length of individual myelin internodes is much larger (on the order of 150 μm). Thus, our measurements of the percent of sheaths with outfoldings in a single EM section (Fig. 2b, c) grossly underestimates the actual percentage of sheaths that have outfoldings^17^. To estimate the actual outfolding frequency in OL-CalEx optic nerves, we used published values for average sheath length in the developing optic nerve^49,105^ combined with our own measurements of the length along a myelin internode an outfolding remains attached. Our quantifications are as follows (see also Table 1 below):

#### Sheath lengths (**L**)

In wild-type optic nerves, the average myelin sheath length has been reported to be ~150 μm in early developmental stages (P30-60)^49,105^. In our sparse-labeling experiments in the spinal cord, we found that myelin sheaths in CalEx are ~70% the length of wild-type sheaths, so we estimate that myelin sheath length (**L**) in CalEx optic nerves is ~105 μm.

#### Outfolding connection length

We used our 3D SEM reconstructions to measure the average outfolding connection length (OCL, equal to the longitudinal length along a myelin sheath with direct connection to an outfolding). We measured *n* = 16 outfoldings (where the entire connection to the myelin sheath was located within the 3D volume), by counting the number of sections we could definitively trace an outfolding back to an axon and multiplying this number by the section thickness (55 nm per section) from our 3DSEM data. This produced the OCL:

(1) OCL = # of sections an outfolding was connected to an axon • section thickness (55 nm)

On average, outfoldings extended **2.9** μm along the longitudinal length of a sheath in OL-CalEx mice (i.e., OCL = 2.9 μM).

The average percentage of a sheath’s length connected to an outfolding (**CP**, connection percentage) equals OCL / L • 100. Plugging in the numbers for L and OCL above (see calculations in Table 1), we conclude that an average outfolding is connected to its corresponding myelin sheath along only **~2.8%** (2.9 μm / 105 μm • 100) of the sheath’s length.

#### Estimating actual percentage of sheaths with outfoldings

To estimate the actual percentage of OL-CalEx sheaths with outfoldings, we must multiply the measured percent of sheaths with outfoldings per section by a multiplication factor of 35.7 (100%/2.8%). The percent of OL-CalEx sheaths with long outfoldings observed in ultrathin sections at P21 was 4.2% (Fig. 2c). Thus, the estimated percent of sheaths with long outfoldings in OL-CalEx mice is *~149.9%* (4.2% x 35.7), meaning that every OL-CalEx myelin sheath is likely to have between 1 and 2 long outfoldings somewhere along its length. Note that in our 3D EM reconstructions both sheaths have multiple separate outfoldings (Fig. 2d), consistent with this estimation.

### Statistics and analysis

Analysis of data in this paper was conducted blind to genotype and experimental condition (i.e. construct ID). Microsoft Excel 16.62.1 and GraphPad Prism 9.0 software were used for data analysis and statistical testing. Descriptive statistics (mean, SEM, and biological replicates) and statistical testing were reported in Figure legends. To determine the method of statistical testing, q-q plots of each dataset were made to visualize whether the datasets were normally or non-normally distributed. For experiments performed with primary oligodendrocytes, statistics were performed on the means from biological replicates (cells from different cell preps); each mean was calculated from the technical replicates within a biological replicate. This distribution of data is represented using Superplots^106^ throughout the paper; each gray dot represents a value from a single cell, while colored dots represent means from biological replicates.

### Reporting summary

Further information on research design is available in the Nature Portfolio Reporting Summary linked to this article.

### Supplementary information

Supplementary Information

Description of Additional Supplementary Files

Supplementary Video 1

Supplementary Video 2

Supplementary Video 3

Reporting Summary

### Source data

Source Data

## Data Availability

The data used in this study are available in the FigShare database under the following accession code 10.6084/m9.figshare.24480973. Source data are provided with this paper.
