# Supplementary material for: Oligodendrocyte calcium signaling promotes actin-dependent myelin sheath extension
Source: Nat Commun. 2024 Jan 4;15:265. doi: 10.1038/s41467-023-44238-3 (PMC10767123; doi:10.1038/s41467-023-44238-3)
Supplement: Supplementary file 1 — Supplementary Information [file 41467_2023_44238_MOESM1_ESM.pdf]

Supplementary Materials

Supplementary materials for *Oligodendrocyte calcium signaling promotes actin-dependent myelin sheath extension.*

Manasi Iyer, Husniye Kantarci, Madeline H. Cooper, Nicholas Ambiel, Sammy Weiser Novak, Leonardo R. Andrade, Mable Lam, Graham Jones, Alexandra E. Münch, Xinzhu Yu, Baljit S. Khakh, Uri Manor, & J. Bradley Zuchero

Contents include the following:

- Supplementary Figures and Figure Legends for Supplementary Figs. 1-10
- Supplementary Table 1 and Table Legend

Supplementary Fig. 1

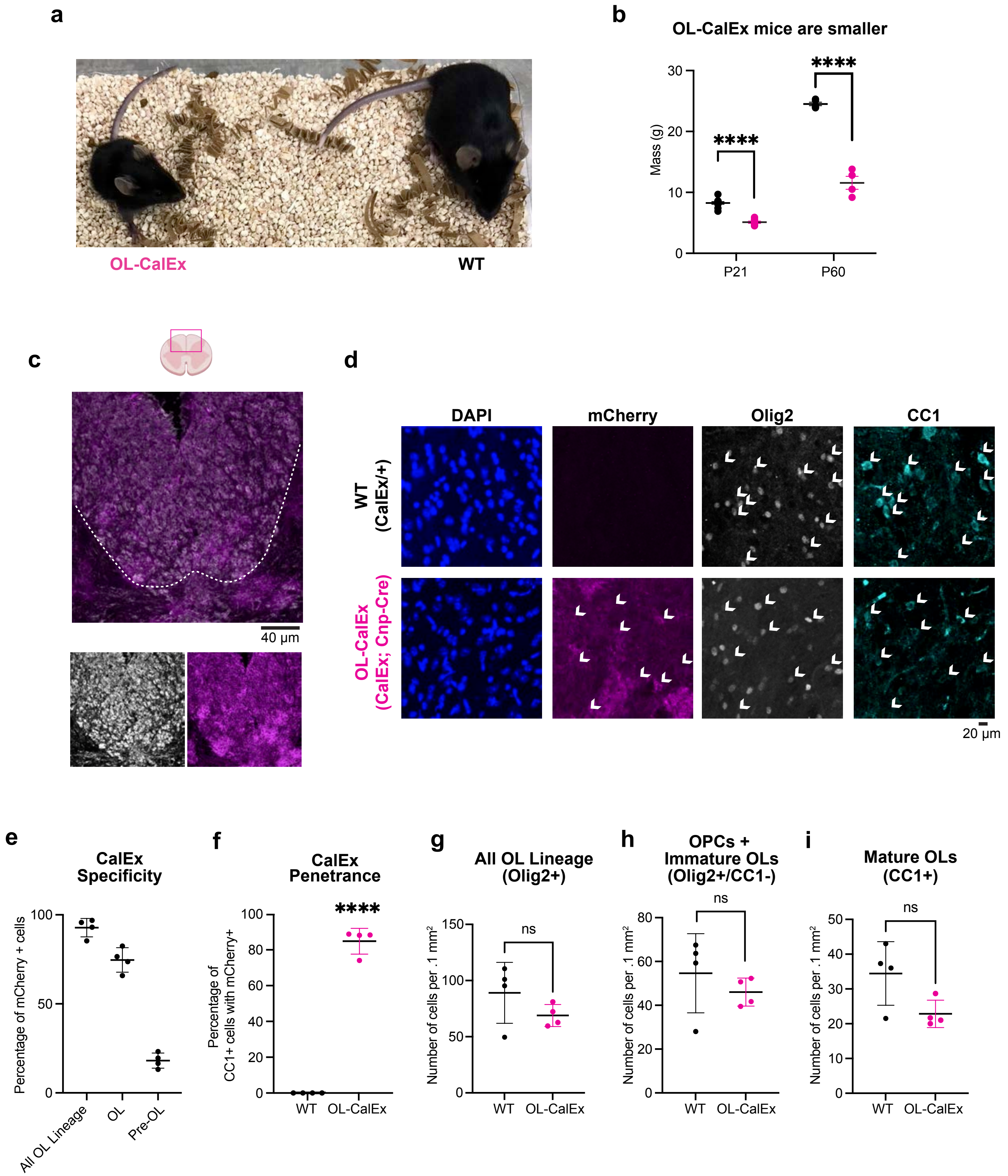

Supplementary Figure 1. CalEx is expressed in OL lineage cells and does not have overt effects on OL lineage numbers, related to Figure 1.

(a) (right) and OL-CalEx (left) littermates at P60.

(b) Body weight for WT and OL littermates at P21 and P60. Statistical measurements (p-values) were determined by unpaired, two-tailed t-tests; p = 0.0001.

(c) Immunolabeling of P21 spinal cord cross section from OL-CalEx co-stained with MBP (grey) and mCherry (magenta). Dotted line denotes the border between white (dense, MBP rich regions on the edges of white matter) and grey matter. Scale bar, 40 μm. Created with Biorender.

(d) Confocal representative micrograph of control (top row) and OL-CalEx (bottom row) P21 dorsal spinal cords stained with DAPI (blue), mCherry (magenta), Olig2 (grey), and CC1 (teal). White arrows point out mature oligodendrocytes (Olig2+, CC1+ cells). Scale bar, 20 μm.

(e) Quantification of CalEx specificity by counting the percentage of mCherry+ cells with the following cell markers (average +/- SEM): Olig2 (all oligodendrocyte lineage cells), Olig2+/CC1+ (mature oligodendrocytes), and Olig2+/CC1- (immature oligodendrocytes and OPCs). N = 4 biological replicates.

(f) Quantification of CalEx penetrance by counting the percentage of mature CC1+ cells contained mCherry (average +/- SEM) in WT and OL-CalEx littermates. N = 4 biological replicates. Statistical measurement (p-value) was determined by an unpaired, two-tailed t-test; ****p = 0.0001.

(g) Quantification of number of all oligodendrocyte lineage cells per field of view in WT and OLCalEx littermates. Three fields of view per biological replicate, N = 4 biological replicates. Statistical measurement (p-value) was determined by an unpaired, two-tailed t-test; n.s. not significant.

(h) Quantification of number of immature oligodendrocytes and OPCs per field of view in WT and OL-CalEx littermates. Three fields of view per biological replicate, N = 4 biological replicates. Statistical measurement (p-value) was determined by an unpaired, two-tailed t-test; n.s. not significant.

(i) Quantification of number of mature oligodendrocytes per field of view in WT and OL-CalEx littermates. Three fields of view per biological replicate, N = 4 biological replicates. Statistical measurement (p-value) was determined by an unpaired, two-tailed t-test; n.s. not significant.

Supplementary Fig. 2

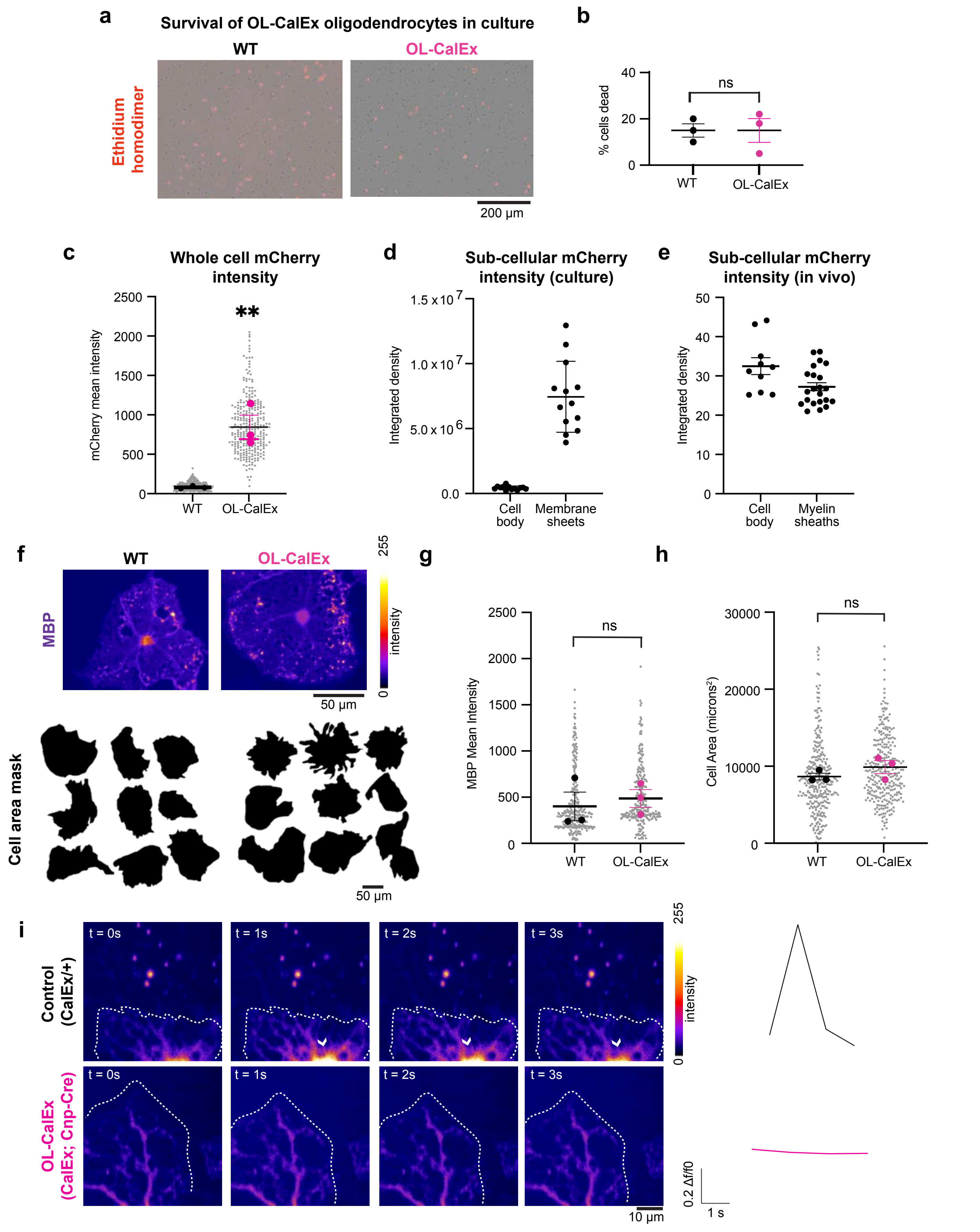

Supplementary Figure 2. Characterization of CalEx in primary cultured oligodendrocytes, related to Figure 1.

(a) Representative micrograph of WT and OL-CalEx cells. Cells visualized using brightfield and ethidium homodimer is depicted in red overlay. Scale bar, 200 μm

(b) Quantification of percentage of dead cells (Average +/- SEM, 3 FOV per condition, N = 3 biological replicates/preps) in WT and OL-CalEx

(c) Quantification of mCherry fluorescence in WT versus OL-CalEx cells. N = 3 biological replicates. Statistical measurement (p-value) was determined by an unpaired, two-tailed t-test; **p = 0.0074.

(d) Quantification of subcellular mCherry fluorescence in OL-CalEx cells in oligodendrocyte cell bodies versus membrane sheets.

(e) Quantification of subcellular mCherry fluorescence in OL-CalEx oligodendrocytes in vivo in cell bodies versus myelin sheaths.

(**f**) (top) Representative micrograph of myelin basic protein staining in (top) WT and (bottom) OL-CalEx oligodendrocytes. (bottom) Representative cell area masks for WT or OL-CalEx cells. Scale bar, 50 μm.

(g) Quantification of myelin basic protein mean intensity (Average +/- SEM) in WT and OLCalEx oligodendrocytes. Statistical measurement (p-value) was determined by an unpaired, two-tailed t-test; n.s. not significant.

(h) Quantification of cell area (Average +/- SEM) for WT or OL-CalEx cells. Statistical measurement (p-value) was determined by an unpaired, two-tailed t-test; n.s. not significant. (g)

(i) (left) Representative micrograph and traces of calcium transient in WT oligodendrocyte process (top) and lack of calcium fluctuations in OL-CalEx oligodendrocyte (bottom). White arrowhead points to calcium transient occurring in the soma of the cultured oligodendrocyte. White dotted lines denote cell borders. (right) Traces from example micrographs.

Supplementary Fig. 3

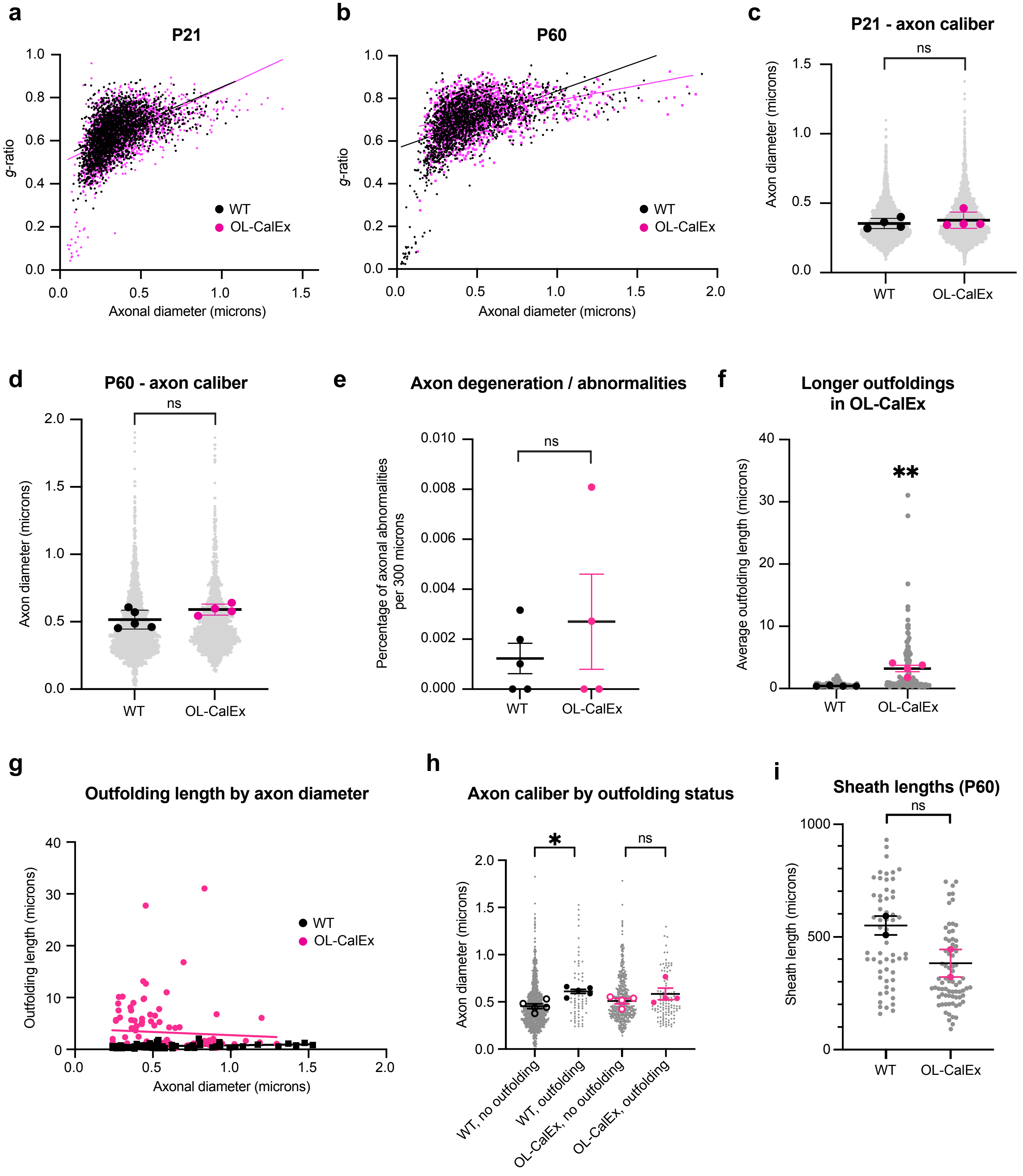

Supplementary Figure 3. Additional ultrastructural analysis of OL-CalEx and WT littermate mice, related to Figure 1.

(a) Distribution of *g*-ratio versus axonal caliber in WT vs OL-CalEx optic nerves at P21.

(b) Distribution of *g*-ratio versus axonal caliber in WT vs OL-CalEx optic nerves at P60.

(c) Quantification of axonal diameter in P21 optic nerves. Average +/- SEM of WT and OLCalEx littermates. N = 4 biological replicates. Statistical measurement (p-value) was determined by an unpaired, two-tailed t-test; n.s. not significant.

(d) Quantification of axonal diameter in P60 optic nerves. Average +/- SEM of WT N = 5 and OL-CalEx littermates N = 4. Statistical measurement (p-value) was determined by an unpaired, two-tailed t-test; n.s. not significant.

(e) Quantification of axonal degeneration or abnormalities in P60 optic nerves. Average +/- SEM of WT N = 5 and OL-CalEx littermates N = 4. Statistical measurement (p-value) was determined by an unpaired, two-tailed t-test; n.s. not significant.

(f) Quantification of outfolding length in P60 optic nerves. Average +/- SEM of WT N = 5 and OL-CalEx littermates N = 4. Statistical measurement (p-value) was determined by an unpaired, two-tailed t-test; **p = 0.0016.

(g) Relationship of outfolding length versus axonal caliber in WT vs OL-CalEx optic nerves at P60.

(h) Quantification of axon caliber of myelinated axons with or without an outfolding in WT and OL-CalEx P60 optic nerves. Average +/- SEM of WT N = 5 and OL-CalEx littermates N = 4. Statistical measurement (p-value) was determined by an unpaired, two-tailed t-test; n.s. not significant.

(i) Quantification of myelin sheath (internode) length from P60 WT and OL-CalEx spinal cords following AAV-mediated sparse labeling (using myelin basic promoter-driven EGFP-caax) as in Fig. 2g-h. Average +/- SEM, N = 2 per genotype. Statistical significance determined by unpaired, two-tailed t-test; n.s. not significant.

Supplementary Fig. 4

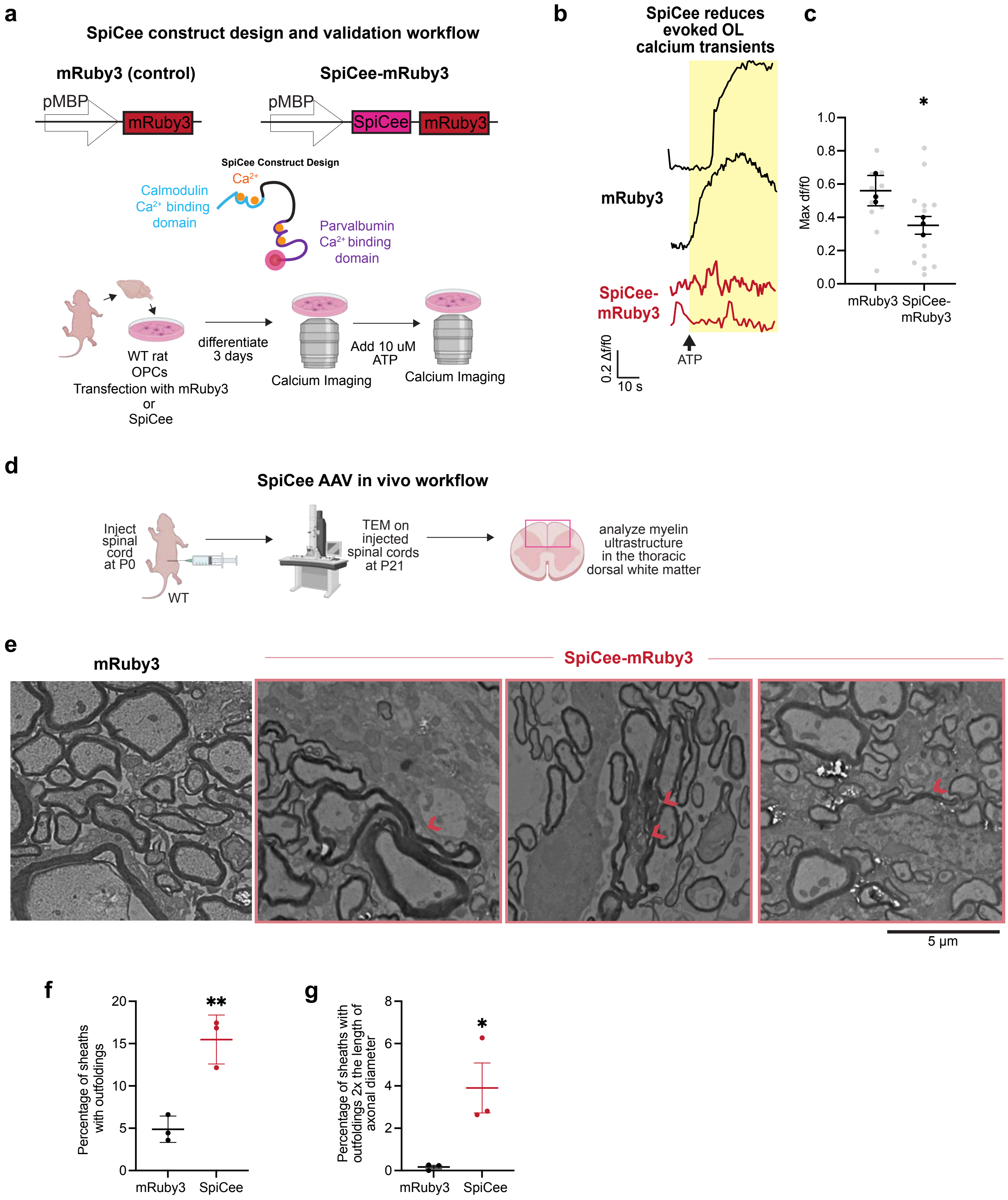

Supplementary Figure 4. Oligodendrocyte-specific expression of SpiCee "calcium sponge" induces outfolding formation in vivo, related to Figure 2.

(a) Construct design for MBP promoter driven mRuby3 (control, left) and MBP promoter driven SpiCee mRuby3 (right). SpiCee is made up of a low-Ca^{2+}-affinity calmodulin domain and a high Ca^{2+}-affinity parvalbumin domain joined by a floppy linker and mRuby3 downstream to label cells that express SpiCee. SpiCee was validated by transfection of OPCs with either mRuby3 or SpiCee-mRuby3. Cells differentiated for 3 days before performing calcium imaging. To stimulate calcium transients in oligodendrocytes, ATP was added to the cells.

(b) Example traces from (top) or SpiCee transfected oligodendrocytes. Arrow indicates addition of ATP.

(c) Quantification of maximum amplitude of calcium transients after ATP addition. Average +/- SEM, N = 3 technical replicates. P-value determined by unpaired, two-tailed Student's t-test; *p = 0.02.

(d) Virus encoding for SpiCee-mRuby3 or mRuby3 was injected into P0 mouse pups. Injected spinal cords were harvested at P21 and processed for transmission electron microscopy.

(e) Example TEM micrograph of mRuby3 injected (left) or SpiCee-mRuby3 injected (right) P21 spinal cords. Red arrows point to outfoldings. Scale bar, 5 μm.

(f) Quantification of percentages of myelin sheaths with outfoldings at P21 in e. Average +/- SEM, P21, N = 3. p-value determined by unpaired, two-tailed Student's t-test; **p = 0.0050.

(g) Quantification of percentages of myelin sheaths with extremely-long outfoldings (defined as an outfolding greater than 2x the diameter of the axons) in e. Average +/- SEM, P21, N = 3. pvalue determined by unpaired, two-tailed Student's t-test; *p = 0.0345.

Supplementary Fig. 5

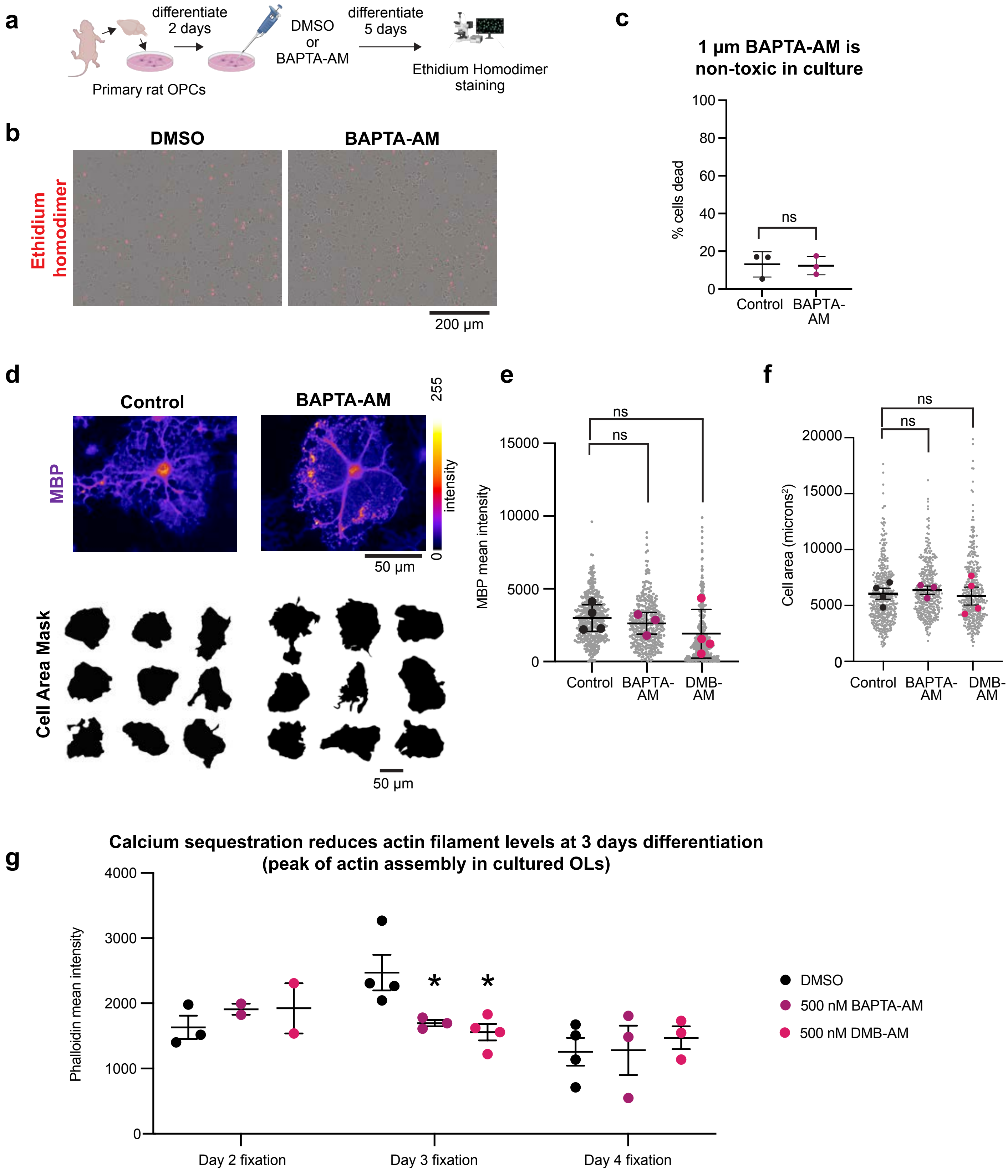

Supplementary Figure 5. Additional data on BAPTA/DMB treatment of primary cultured oligodendrocytes, related to Figure 3.

(a) Experimental set up for determining percentage of dead cells in BAPTA treated cells. Primary rat OPCs were differentiated for two days and then treated overnight with either DMSO or BAPTA-AM. Cell media was then replaced with normal OL media and cells continued differentiating for 5 days before treatment with ethidium homodimer to label dead cells. Scale bar, 200 μm.

(b) Representative micrograph of DMSO and BAPTA treated cells. Cells visualized using brightfield and ethidium homodimer is depicted in red overlay.

(c) Quantification of percentage of dead cells (Average +/- SEM, 3 FOV per condition, N = 3 biological replicates/preps) in DMSO-treated and BAPTA-treated cells; P-value determined by unpaired, two-tailed Student's t-test; n.s., not significant.

(d) Representative micrograph of myelin basic protein staining (top) and (bottom) cell area masks for cells treated with DMSO or BAPTA. Scale bar, 50 μm.

(e) Quantification of myelin basic protein mean intensity (Average +/- SEM) in DMSO treated, BAPTA-AM treated, or DMB-AM treated. P-value determined by one way ANOVA; n.s., not significant.

(f) Quantification of cell area (Average +/- SEM) in DMSO treated, BAPTA-AM, and DMB-AM treated. P-value determined by unpaired, two-tailed Student's t-test; n.s., not significant.

(g) Quantification of phalloidin mean intensity (Average +/- SEM) across three days of oligodendrocyte differentiation; P-value determined by one-way ANOVA;*p = 0.0188.

Supplementary Fig. 6

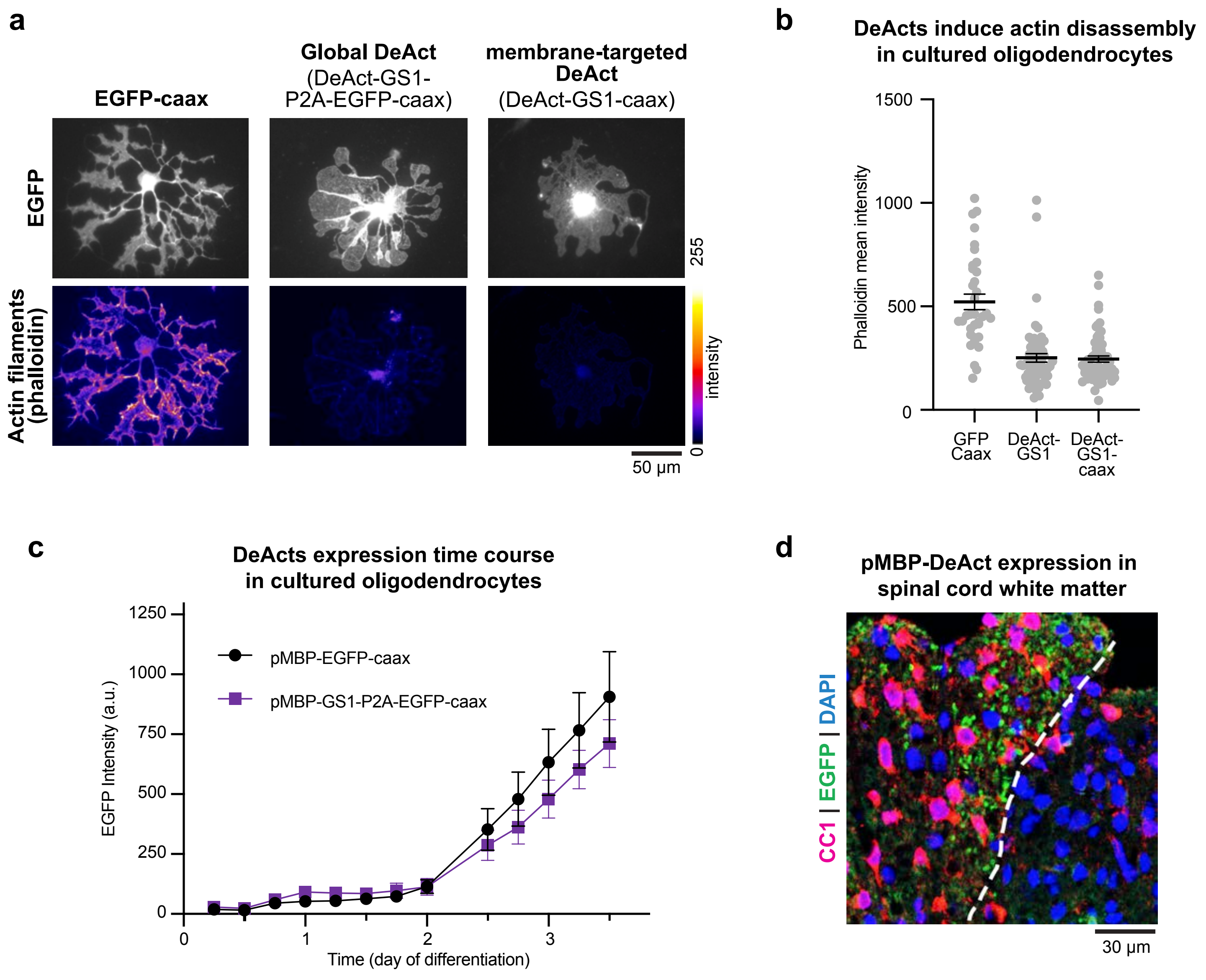

Supplementary Figure 6. Validation of DeActs in cultured and in vivo oligodendrocytes, related to Figure 4.

(**a**) Representative micrographs of phalloidin staining (bottom) and EGFP expression (top) for cells transfected with either EGFP-caax, DeAct-GS1, or DeAct-GS1-caax. Scale bar, 50 μm.

(**b**) Quantification of phalloidin mean intensity (Average +/- SEM) in EGFP-caax, DeAct-GS1 or DeAct-GS1-caax.

(c) Time course of expression of MBP promoter (pMBP)-driven expression of EGFP-caax and GS1-P2A-EGFP-caax.

(d) Confocal micrograph of spinal cord cross section injected with EGFP-caax. CC1: Magenta, EGFP: Green, DAPI: Blue. Dotted line denotes the border of the white matter and grey matter. Scale bar, 30 μm.

Supplementary Fig. 7

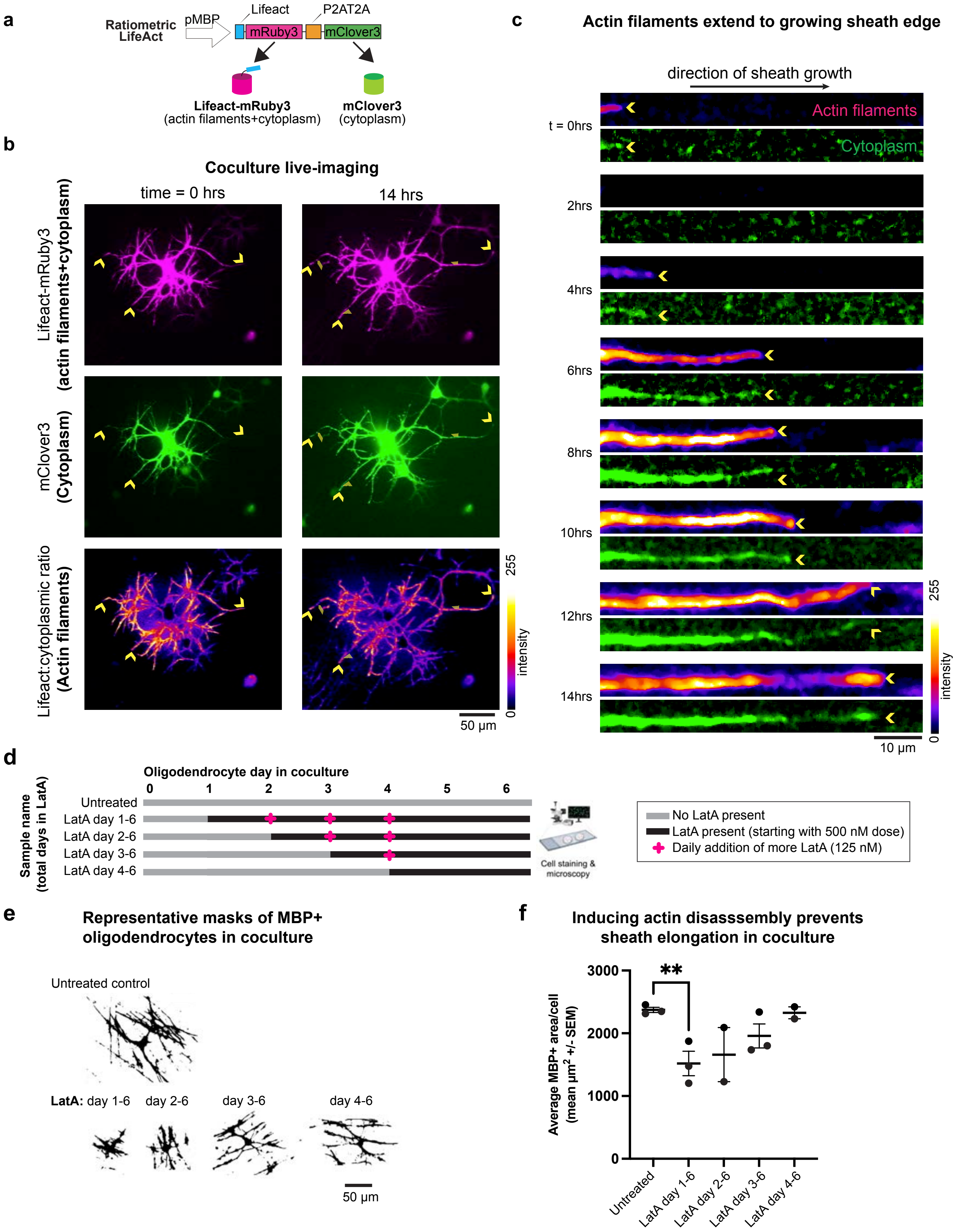

Supplementary Figure 7. Actin filament assembly drives longitudinal grown of nascent myelin sheaths in myelinating cocultures.

(a) Schematic showing design of Ratiometric Lifeact construct (RMLA; see Methods) driven by the MBP promoter. After translation, the P2AT2A peptide causes separation of Lifeact-mRuby3 (binds actin filaments) from mClover3 (marks cytoplasm/nucleoplasm).

(b) Representative stills of 1st (0hrs) and last (14hrs) frame of live-cell video of day 4 oligodendrocyte extending sheaths along retinal ganglion cell (RGC) axons. Oligodendrocytes express Ratiometric Lifeact. Arrows indicate representative sheath edges, with faint arrows indicating location of sheath edge at start of imaging. LifeAct-mRuby3 (top row) and mClover3 (middle row) channels shown are artificially corrected for differences in expression levels over time to highlight sheath edges. Bottom row shows ratiometric image which highlights actin filaments (note the cell body and nucleus—which has a large volume as marked by mClover3 above but low levels of actin filaments—is devoid of ratiometric signal).

(c) Top process from (a) zoomed in and straightened to visualize sheath edge at each time point. Aligned ratiometric Lifeact signal (labeling actin filaments; Fiji Fire LUT) and mClover3 (labeling cytoplasm; grey) at the tip of the dynamic sheath. Scale bar, 10 µm.

(d) Experimental timeline for myelinating cocultures. Oligodendrocytes grown in coculture on dense beds of RGC axons were treated with Latrunculin A (LatA) at different time points, grown until day 6, then fixed and stained for MBP to image and quantify sheaths.

(e) Representative masks of individual cocultured oligodendrocytes that were either untreated (top) or treated with LatA for various lengths of time (longest treatment time to shortest from left to right). Scale bar, 50 µm.

(f) Quantification of average total myelin sheath length per oligodendrocyte (Average +/- SEM) from experiment shown in (c) & (d). N = 2-3 biological replicates per time point; P-value determined by unpaired t-test comparing each treated group to untreated **p = 0.0128.

Supplementary Fig. 8

a **Initial attempts to stabilize actin filaments using StablAct construct**

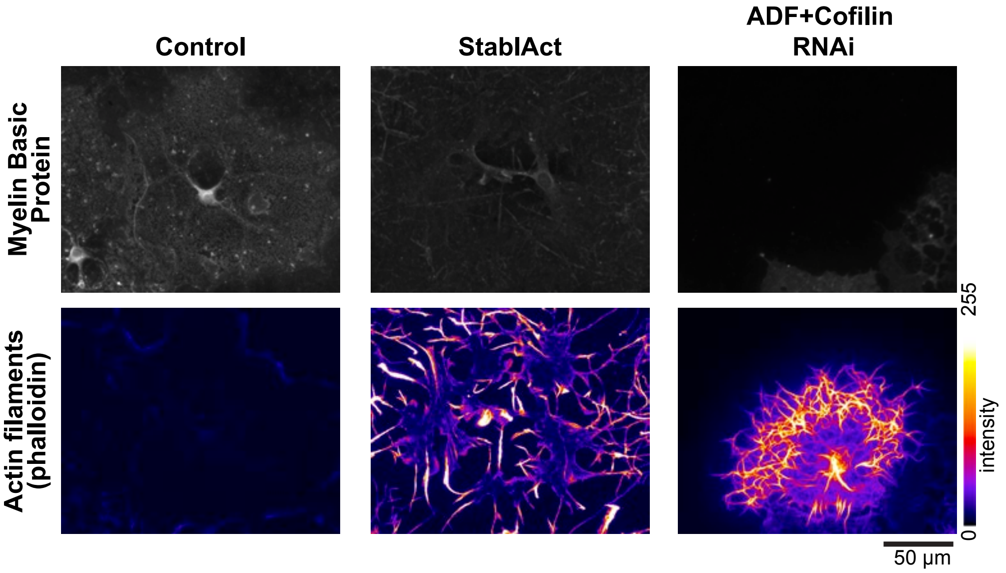

EM analysis of Ezrin(abd*) expression in vivo

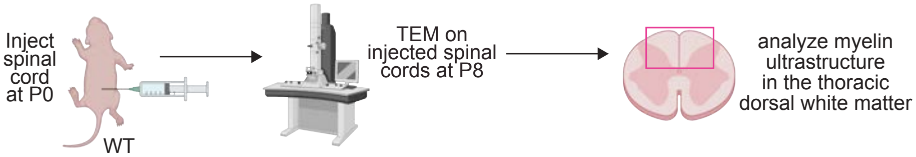

b

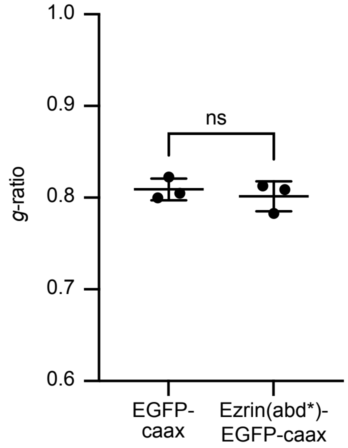

c

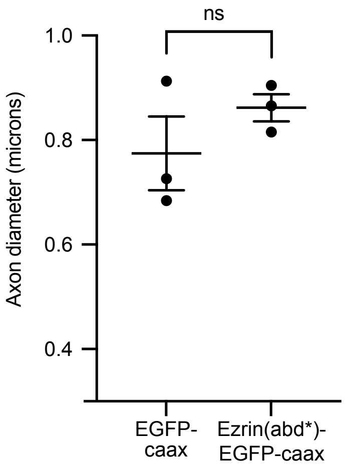

Supplementary Figure 8. Validation and characterization of actin stabilizing tools in oligodendrocytes.

(a) Representative micrograph of phalloidin staining and myelin basic protein for cells transfected with control constructs, prototype “StablAct” actin-stabilizing construct, or ADF/cofilin double RNAi. Scale bar, 50 μm.

(b) Quantification of myelin thickness via *g*-ratio from electron microscopy of EGFP-caax or Ezrin(abd*)-EGFP-caax. Average +/- SEM, N = 3. Statistical significance determined by unpaired, two-tailed Student’s t-test; n.s., not significant.

(c) Quantification of axonal caliber from electron microscopy of EGFP-caax or Ezrin(abd*)EGFP-caax. Average +/- SEM, N = 3. Statistical significance determined by unpaired, two-tailed Student’s t-test; n.s., not significant.

Supplementary Fig. 9

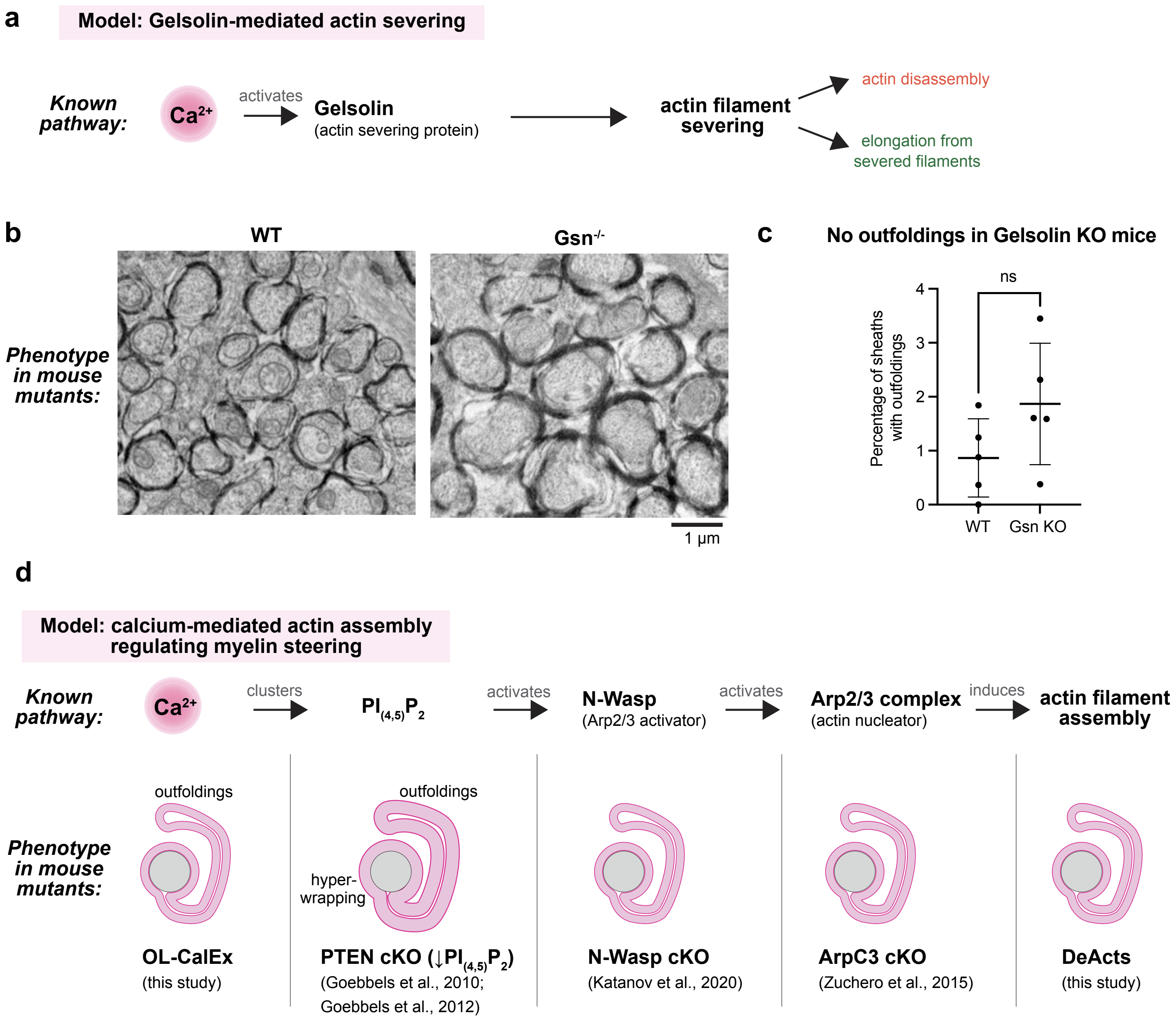

Supplementary Figure 9. Models for how calcium may regulate actin severing in developing myelin sheaths, related to Figure 6.

(a) Model for how calcium may regulate actin severing in oligodendrocytes to steer myelin membrane growth. Calcium is known to activate actin-severing protein gelsolin, which promotes both actin disassembly as well as formation of new filaments from newly-generated barbed ends.

(b) Transmission electron microscopy of WT (left) and Gsn KO (right) optic nerve sections. Scale bar, 1 μm.

(c) Quantification of myelin sheaths with outfoldings in e. Average +/- SEM, N = 5. P-value determined by unpaired, two-tailed t-test; n.s., not significant.

(d) Molecular mechanism for how calcium may regulate actin assembly. Calcium is known to cluster $PI_{(4,5)}P_2$, which in turn activates N-WASP to activate the Arp2/3 complex to induce actin filament assembly. Conditional knockout of any component of this pathway leads to increased outfoldings.

Supplementary Fig. 10

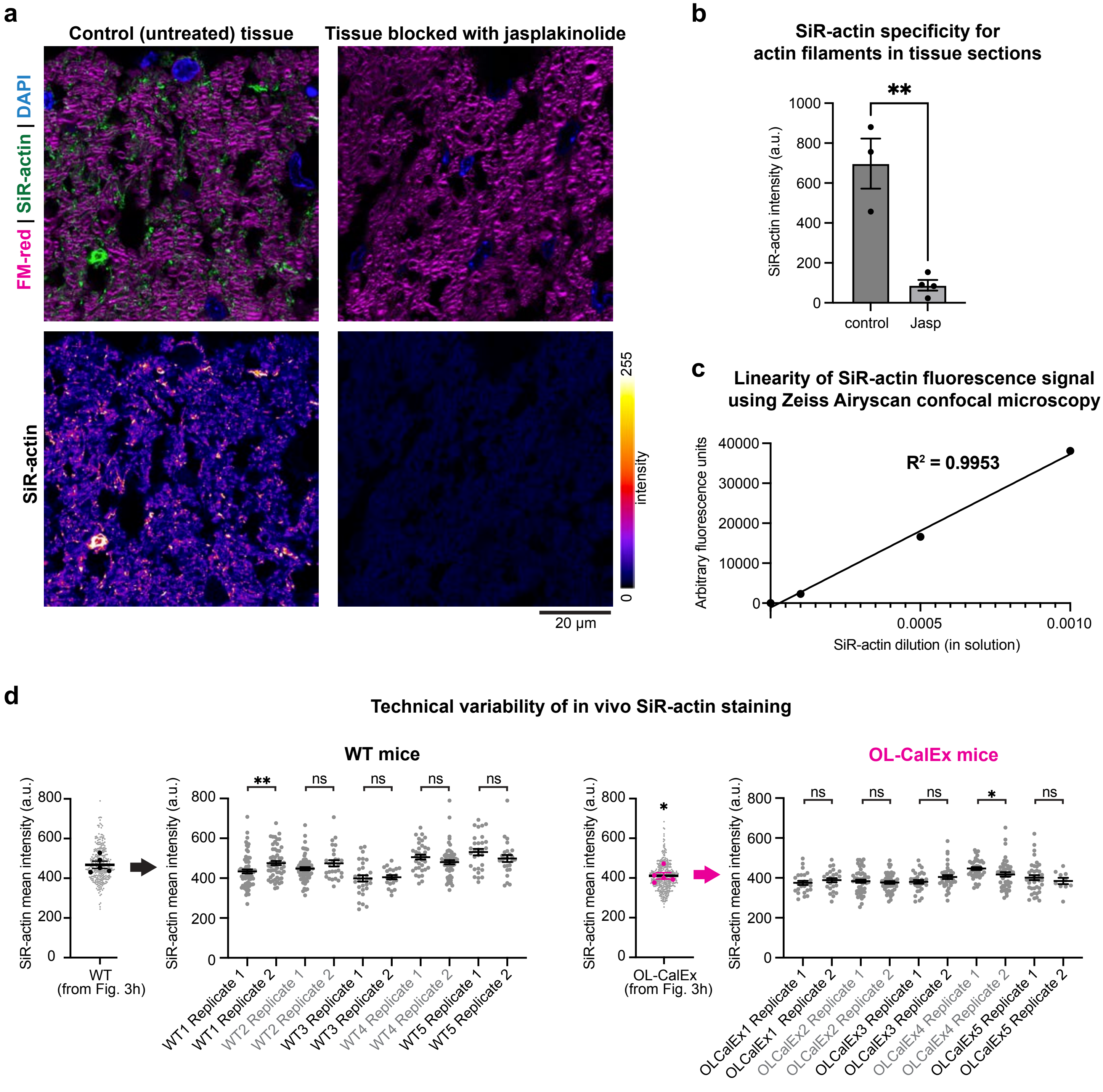

Supplementary Figure 10. Controls for SiR-actin staining of tissue.

(**a**) To assess specificity of SiR-actin tissue for actin filaments, fixed and permeabilized P20 spinal cord cryosections were incubated with staining solution containing Fluoromyelin red (FMred; magenta), SiR-actin (green), and DAPI (blue) (all as in Fig. 3g) with or without 5 µM jasplakinolide, a high-affinity and specific actin filament binding drug that binds to the same site on actin filaments as SiR-actin[56]. Representative images show all three-color channels (top row) or single-channel images of SiR-actin displayed using the Fire LUT in Fiji (bottom row).

(**b**) Quantification of background-subtracted mean SiR-actin fluorescence intensity in tissues shown in (b). Average +/- SEM, tissues from N = 3-4 mice per group. P-value determined by unpaired, two-tailed t-test; **p = 0.0026.

(c) We prepared a dilution series of SiR-actin dye in PBS, then imaged each solution in a custom-built flow chamber using Zeiss Airyscan confocal microscopy with identical acquisition parameters as in Fig. 3 g-h (see Methods). Quantification reveals a linear relationship between SiR-actin concentration and fluorescence, validating this imaging workflow for quantitative fluorescence measurements.

(d) Data from Fig. 3h, replotted to split apart individual technical and biological replicates. For both WT and OL-CalEx mice, SiR-actin in spinal cord myelin sheaths of N=5 mice were imaged. For each biological replicate, two technical replicates (separate tissue sections) were imaged. Light grey dots show SiR-actin mean intensity measurements in individual myelin sheaths, while the error bars show mean and SEM for each technical replicate. P-values compare all individual sheath measurements between technical replicates using T-tests, and show that in most cases, technical replicates are not significantly different (but occasionally are, highlighting why we need to average more than one technical replicate to get an accurate mean value for each biological replicate; see Methods). **p = 0.0025, *p = 0.0230.

Paper	PMID (Figure) Ref. #	Model system	Manipulation	Control sheath length	Experimental sheath length	Percent change in sheath length
Brown 2021	33478987 (Fig. 7E) Ref. 66	Zebrafish spinal cord, 48-72hr post-fertilization	PAK1 inhibitor	~16 µm	~11 µm	**-31.2%**
**This study**	N/A (Fig. 2h)	Mouse spinal cord, P21	**OL-CalEx expression**	264 µm	183 µm	**-30.7%**
Lam 2022	36151203 (Fig. 4g) Ref. 15	Mouse cortex, P12	OL botulinum toxin expression	54.2 µm	39.6 µm	**-26.9%**
Swire 2021	34341156 (Fig. 5G) Ref 67	Mouse spinal cord, P50	HCN2 cKO	~424 µm	~315 µm	**-25.7%**
Lam 2022	36151203 (Fig. S9i) Ref. 15	Mouse spinal cord, P12	OL expression of dn-VAMP2	110 µm	84 µm	**-23.6%**
**This study**	N/A (Fig. 4h)	Mouse spinal cord, P12	**DeAct-GS1 expression**	234 µm	185 µm	**-20.9%**
Bacmeister 2022	36180791 (Fig. 2c) Ref. 3	Mouse cortex, 21 days post motor learning	Before/after learning	~57 µm	~47 µm	**-17.5%**

Supplementary Table 1. Myelin sheath length reductions in published studies compared to OL-CalEx and DeAct-GS1. We used www.graphreader.com to approximate the sheath length changes reported in the publications shown in the table, and calculated percent change in sheath length for allperturbations listed (negative value means the sheaths shortened). Findings from OL-CalEx and DeAct-GS1 (reported here) are highlighted in blue.
