# Supplementary material for: Oligodendrocyte calcium signaling promotes actin-dependent myelin sheath extension
Source: Nat Commun. 2024 Jan 4;15:265. doi: 10.1038/s41467-023-44238-3 (PMC10767123; doi:10.1038/s41467-023-44238-3)
Supplement: Supplementary file 2 — Description of Additional Supplementary Files [file 41467_2023_44238_MOESM2_ESM.docx]

**Description of Additional Supplementary Files**

**Supplementary Video 1:**

Calcium signaling in WT and OL-CalEx primary oligodendrocytes,

related to Figure 1. (right) Fluo4-AM loaded primary WT oligodendrocyte at day 3 of

differentiation. (left) Fluo4-AM loaded primary OL-CalEx oligodendrocyte at day 3 of

differentiation.

**Supplementary Video 2:**

3-dimensional reconstruction of two myelinated axons with

outfoldings from OL-CalEx optic nerve, related to Figure 2. Scale: movie dimensions are 7.57 μm (height) x 7.57 μm (width).

**Supplementary Video 3:**

360-degree view of 3-dimensional reconstruction of two myelin

sheaths with outfoldings from OL-CalEx optic nerve, related to Figure 2. Scale: rotating cube shown in movie is 1.4 μm on each side.
