# Supplementary material for: Oligodendrocyte calcium signaling promotes actin-dependent myelin sheath extension
Source: Nat Commun. 2024 Jan 4;15:265. doi: 10.1038/s41467-023-44238-3 (PMC10767123; doi:10.1038/s41467-023-44238-3)
Supplement: Supplementary file 6 — Reporting Summary [file 41467_2023_44238_MOESM6_ESM.pdf]

nature portfolio

Corresponding author(s): J. Bradley Zuchero

Last updated by author(s): November 28. 2023

Reporting Summary

Nature Portfolio wishes to improve the reproducibility of the work that we publish. This form provides structure for consistency and transparency in reporting. For further information on Nature Portfolio policies, see our Editorial Policies and the Editorial Policy Checklist.

Statistics

For all statistical analyses, confirm that the following items are present in the figure legend, table legend, main text, or Methods section.

n/a	Confirmed	
☐	☒	The exact sample size (*n*) for each experimental group/condition, given as a discrete number and unit of measurement
☐	☒	A statement on whether measurements were taken from distinct samples or whether the same sample was measured repeatedly
☐	☒	The statistical test(s) used AND whether they are one- or two-sided<br>*Only common tests should be described solely by name; describe more complex techniques in the Methods section.*
☐	☒	A description of all covariates tested
☐	☒	A description of any assumptions or corrections, such as tests of normality and adjustment for multiple comparisons
☐	☒	A full description of the statistical parameters including central tendency (e.g. means) or other basic estimates (e.g. regression coefficient) AND variation (e.g. standard deviation) or associated estimates of uncertainty (e.g. confidence intervals)
☐	☒	For null hypothesis testing, the test statistic (e.g. *F*, *t*, *r*) with confidence intervals, effect sizes, degrees of freedom and *P* value noted<br>*Give P values as exact values whenever suitable.*
☒	☐	For Bayesian analysis, information on the choice of priors and Markov chain Monte Carlo settings
☒	☐	For hierarchical and complex designs, identification of the appropriate level for tests and full reporting of outcomes
☒	☐	Estimates of effect sizes (e.g. Cohen's *d*, Pearson's *r*), indicating how they were calculated

Our web collection on statistics for biologists contains articles on many of the points above.

Software and code

Policy information about availability of computer code

Data collection	Zen Blue 2.6 (Zeiss), IncuCyte Zoom (Essen Biosciences), Gatan OneView (Gatan)
Data analysis	Fiji/Image J 1.0 (NIH), GraphPad Prism 9.10 (216), Excel (Microsoft)

For manuscripts utilizing custom algorithms or software that are central to the research but not yet described in published literature, software must be made available to editors and reviewers. We strongly encourage code deposition in a community repository (e.g. GitHub). See the Nature Portfolio guidelines for submitting code & software for further information.

Data

Policy information about availability of data

All manuscripts must include a data availability statement. This statement should provide the following information, where applicable:

- Accession codes, unique identifiers, or web links for publicly available datasets
- A description of any restrictions on data availability
- For clinical datasets or third party data, please ensure that the statement adheres to our policy

Data availabiilty section outlines how source data can be accessed.

The data used in this study are available in the FigShare database under the following accession code https://doi.org/10.6084/m9.figshare.24480973. All correspondence and requests for materials should be addressed to J.B.Z.

Research involving human participants, their data, or biological material

Policy information about studies with human participants or human data. See also policy information about sex, gender (identity/presentation), and sexual orientation and race, ethnicity and racism.

Reporting on sex and gender	The present study does not use human data.
Reporting on race, ethnicity, or other socially relevant groupings	The present study does not use human data.
Population characteristics	The present study does not use human data.
Recruitment	The present study does not use human data.
Ethics oversight	The present study does not use human data.

Note that full information on the approval of the study protocol must also be provided in the manuscript.

Field-specific reporting

Please select the one below that is the best fit for your research. If you are not sure, read the appropriate sections before making your selection.

[x] Life sciences [ ] Behavioural & social sciences [ ] Ecological, evolutionary & environmental sciences

For a reference copy of the document with all sections, see nature.com/documents/nr-reporting-summary-flat.pdf

Life sciences study design

All studies must disclose on these points even when the disclosure is negative.

Sample size	Sample size was calculated using: www.stat.ubc.ca/~rollin/stats/ssize/n2.html. We calculated sample sizes using pilot studies to determine the minimal number of mice needed for each experiment (power=0.8, alpha=0.05). For electron microscopy, based on this and our/other's published work in the field (e.g. PMIDs: 26166300, 36151203, 30551998, 24439382), we predetermined that we would use N=5 mice to test our major hypotheses or N=3 mice for orthogonal confirmation of major results.
Data exclusions	No data have been excluded from the present study.
Replication	All biological replicates are reported in the figure legends. CNS tissues for immunohistochemistry and EM analysis were collected from 3-5 different animals of each genotype. 7 mice sparsely injected with AAVs were harvested for P21 sheath length analyses; 4 mice sparsely injected with AAVs were harvested for P60 sheath length analyses.<br><br>For primary mouse cell experiments, mouse cells from 3-4 different brains of each genotype were analyzed. Each biological replicate for cell images represents the mean of all datapoints harvested from a single mouse brain. For primary rat cells, cells were harvested from a single rat brain, and the cells from this brain represented a single biological replicate.
Randomization	For immunohistochemistry, electron microscopy, viability, and primary oligodendrocyte purification, mice of the same genotype were randomly assigned to experiments. Rat and mouse pups were randomly selected for retinal ganglion cell and oligodendrocyte precursor purifications.
Blinding	Image acquisition and analysis for electron microscopy, immunohistochemistry, and cellular assays were performed with the researcher blinded to the genotype.

Reporting for specific materials, systems and methods

We require information from authors about some types of materials, experimental systems and methods used in many studies. Here, indicate whether each material, system or method listed is relevant to your study. If you are not sure if a list item applies to your research, read the appropriate section before selecting a response.

Materials & experimental systems

n/a	Involved in the study
	☒ Antibodies
	☒ Eukaryotic cell lines
☒	Palaeontology and archaeology
	☒ Animals and other organisms
☒	Clinical data
☒	Dual use research of concern
☒	Plants

Methods

n/a	Involved in the study
☒	ChIP-seq
☒	Flow cytometry
☒	MRI-based neuroimaging

Antibodies

Antibodies used	Primary antibodies used in this study were as follows: Rat-anti-MBP (Abcam ab7349; 1:100), Mouse-anti-CC1 (Millipore Sigma Ab-7, #OP80; specific for Quaking 7 protein enriched in oligodendrocytes; see PMID:27454326; 1:500), Goat-anti-Olig2 (Millipore AB9610; 1:500), Rabbit-anti-RFP (Rockland #600-401-379; 1:1000).<br><br>Secondary antibodies used in this study were as follows at a 1:1000 dilution for both tissue and primary cell immunofluorescence: donkey anti-rat Alexa Fluor 594 (Thermo Scientific A-21209), goat anti-rat Alexa Fluor 647 (Thermo Scientific A-21247), donkey anti-mouse Alexa Fluor 488 (Thermo Scientific A-21202), donkey anti-mouse Alexa Fluor 647 (Thermo Scientific A-31571), donkey anti-rabbit Alexa Fluor 594 (Thermo Scientific, A-21207) Alexa Fluor 488 conjugated Phalloidin (Thermo Scientific, A12379).
Validation	Rat-anti-MBP: We previously validated Abcam ab7349 for immunostaining mouse CNS tissues and primary oligodendrocytes using Shiverer (MBP-null) mice (Zuchero et al., 2015; PMID:26166300).<br>Mouse-anti-CC1: This antibody was validated to specifically recognize Quaking 7, a protein highly enriched in differentiated oligodendrocytes, and is commonly used to mark the cell bodies of differentiated oligodendrocytes (Bin et al., 2016; PMID:27454326).<br>Goat-anti-Olig2: Not validated to our knowledge, but cited in 1095 publications and used for immunostaining (https://www.citeab.com/antibodies/224426-ab9610-anti-olig-2-antibody).<br>Rabbit-anti-RFP: We validated this antibody for immunostaining of mCherry by comparing CalEx-mCherry-expressing mice to littermate controls not expressing CalEx-mCherry (Fig. S1, c-d). Also cited in 1260 publications (https://www.citeab.com/antibodies/1908633-600-401-379-anti-rfp-rabbit-antibody-min-x-hu-ms-a?des=60601c11e690e5fc).

Eukaryotic cell lines

Policy information about cell lines and Sex and Gender in Research

Cell line source(s)	Primary cultures of oligodendrocyte precursors and retinal ganglion cells were prepared using Sprague-Dawley rats and C57BL/6 mice (ordered from Charles River) using previously published protocols. For mouse cell preps, 3-4 mouse brains of both sexes were pooled in order to harvest sufficient cells. For rat cell preps, individual rat brains were used to harvest oligodendrocyte precursors and cells from a single rat brain were considered one biological replicate. Cells from both sexes were analyzed for the present study, and because of low sample sizes, we did not test for differences between sexes.
Authentication	Primary oligodendrocytes were stained for MBP Abcam (ab7349 1:100), a marker of oligodendrocyte differentiation.
Mycoplasma contamination	Primary cultures were acutely purified from mice/rats, and were not tested for mycoplasma contamination. We did not conduct any experiments with immortalized cell lines.
Commonly misidentified lines<br>(See ICLAC register)	Commonly misidentified lines were not used in the present study.

Animals and other research organisms

Policy information about studies involving animals; ARRIVE guidelines recommended for reporting animal research, and Sex and Gender in Research

Laboratory animals	Mice were group housed under a standard 12:12 light-dark cycle. Mouse rooms were kept between 18-23 degrees Celcius and 40-60% humidity. Mice were given ad libitum access to food and water. Mice were housed in plastic cages with disposable bedding. All procedures were approved by Stanford APLAC, protocol number 32260.<br><br>Cnp-CRE/+ mice were obtained from Dr. Klaus Nave (Max Planck Institute for Experimental Medicine) and maintained through crossing to C57Bl/6 mice. CalEx mice were obtained from Dr. Baljit Khakh (UCLA). OL-CalEx (CalEx/+; Cnp-CRE/+) strain was obtained by a single cross of a CNP-Cre/+ heterozygote parent to a CalEx/CalEx homozygote parent. All animals from the resulting cross were used for experiments. Sprague-Dawley rats and C57BL/6 mice were ordered from Charles River Laboratories.<br><br>OL-CalEx mice for IHC were harvested at P8 or P21; for EM they were harvested at P8, P21, or P60. OL-CalEx mouse brains were harvested for primary oligodendrocyte cultures at P6. Males and females were pooled becuase no sexual dimorphism for defects

	seen in the OL-CalEx was observed.<br><br>Sprague-Dawley rats and C57Bl/6 mice were harvested at P6 for OPC isolations, and P5 for RGC isolations.
Wild animals	The present study did not use wild animals.
Reporting on sex	Samples were collected from both male and female for all in vivo studies. For in vivo experiments, both sexes were used and data from males and females were pooled for all experiments. We did not test for differences between sexes because of low sample sizes for each sex.
Field-collected samples	The present study did not use field-collected samples.
Ethics oversight	All procedures involving animals were approved by the Institutional Administrative Panel on Laboratory Animal Care (APLAC; Protocol 32260) of Stanford University and followed the National Institutes of Health guidelines.

Note that full information on the approval of the study protocol must also be provided in the manuscript.
